# Solution Synthesis and Characterization of a Long and Curved Graphene Nanoribbon with Hybrid Cove–Armchair–Gulf Edge Structures

**DOI:** 10.1002/advs.202200708

**Published:** 2022-03-24

**Authors:** Lin Yang, Ji Ma, Wenhao Zheng, Silvio Osella, Jörn Droste, Hartmut Komber, Kun Liu, Steffen Böckmann, David Beljonne, Michael Ryan Hansen, Mischa Bonn, Hai I. Wang, Junzhi Liu, Xinliang Feng

**Affiliations:** ^1^ Centre for Advancing Electronics Dresden (cfaed) Department of Chemistry and Food Chemistry Technische Universität Dresden Dresden 01062 Germany; ^2^ Max Planck Institute for Polymer Research Ackermannweg 10 Mainz 55128 Germany; ^3^ Chemical and Biological Systems Simulation Lab Centre of New Technologies University of Warsaw Banacha 2C Warsaw 02–097 Poland; ^4^ Institute of Physical Chemistry Westfal̈ische Wilhelms‐Universitaẗ (WWU) Münster Corrensstraße 28/30 Münster D‐48149 Germany; ^5^ Leibniz‐Institut für Polymerforschung Dresden e.V. Hohe Straße 6 Dresden 01069 Germany; ^6^ Laboratory for Chemistry of Novel Materials Université de Mons Mons B‐7000 Belgium; ^7^ Department of Chemistry and State Key Laboratory of Synthetic Chemistry The University of Hong Kong Pokfulam Road Hong Kong 999077 China; ^8^ Max Planck Institute of Microstructure Physics Weinberg 2 Halle 06120 Germany

**Keywords:** curved, Diels–Alder polymerization, graphene nanoribbon, low bandgap, multi‐edge structure

## Abstract

Curved graphene nanoribbons (GNRs) with hybrid edge structures have recently attracted increasing attention due to their unique band structures and electronic properties as a result of their nonplanar conformation. This work reports the solution synthesis of a long and curved multi‐edged GNR (**cMGNR**) with unprecedented cove–armchair–gulf edge structures. The synthesis involves an efficient A_2_B_2_‐type Diels–Alder polymerization between a diethynyl‐substituted prefused bichrysene monomer (**3b**) and a dicyclopenta[*e*,*l*]pyrene‐5,11‐dione derivative (**6**) followed by FeCl_3_‐mediated Scholl oxidative cyclodehydrogenation of the obtained polyarylenes (**P1**). Model compounds **1a** and **1b** are first synthesized to examine the suitability and efficiency of the corresponding polymers for the Scholl reaction. The successful formation of **cMGNR** from polymer **P1** bearing prefused bichrysene units is confirmed by FTIR, Raman, and solid‐state NMR analyses. The cove‐edge structure of the **cMGNR** imparts the ribbon with a unique nonplanar conformation as revealed by density functional theory (DFT) simulation, which effectively enhances its dispersibility in solution. The **cMGNR** has a narrow optical bandgap of 1.61 eV, as estimated from the UV–vis absorption spectrum, which is among the family of low‐bandgap solution‐synthesized GNRs. Moreover, the **cMGNR** exhibits a carrier mobility of ≈2 cm^2^ V^−1^ s^−1^ inferred from contact‐free terahertz spectroscopy.

## Graphical abstract

A curved multi‐edged graphene nanoribbon (cMGNR) with an unprecedented cove–armchair–gulf edge topology is synthesized through the efficient A_2_B_2_‐type Diels–Alder polymerization followed by the Scholl reaction. The resultant cMGNR possesses an average length of sub‐hundred nanometer and a narrow optical bandgap, making it a promising candidate for the fabrication of single GNR‐based nanoelectronic devices.

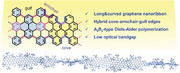

## 1 Introduction

Graphene nanoribbons (GNRs) have attracted enormous interest in recent decades due to their potential applications in transistors, photovoltaics and quantum electronic devices.^[^ ^1^ ^]^ The current synthetic methods for GNRs consist of top‐down and bottom‐up approaches.^[^ ^2^ ^]^ In contrast to top‐down methods by cutting graphene or unzipping carbon nanotubes, bottom‐up organic synthesis represents an efficient approach to constructing GNRs with atomic precision.^[^ ^3^ ^]^ To date, the representative bottom‐up synthetic route of GNRs includes the solution‐phase or surface‐assisted polymerization of tailor‐made molecular precursors to form polyarylene polymers followed by the subsequent intramolecular cyclodehydrogenation (or Scholl reaction).^[^ ^3^ ^,‐^ ^4^ ^]^ In particular, the solution‐synthesis protocol has enabled the preparation of large‐scale GNRs with control over the width, length, and edge structure. In addition, the rational geometry control of GNRs by edge topology engineering has recently received considerable attention owing to its high potential for optoelectronic property tuning.^[^ ^5^ ^]^ For example, the introduction of cove‐ or fjord‐type edges on the periphery of GNRs significantly alters their topological conformations and electronic structures. Very recently, we demonstrated that GNRs bearing cove edges exhibit nonplanar geometry with a low bandgap and high charge carrier mobility.^[^ ^5d,e^ ^]^ Moreover, highly twisted GNRs containing fjord edges were recently synthesized, which represent potential candidates as chiral optical materials and chiral catalysts.^[^ ^5c,f^ ^]^ Despite substantial progress toward the synthesis of nonplanar GNRs, strategies for the preparation of long and curved GNRs remain elusive, which hamper their further integration into single GNR‐based devices.^[^ ^6^ ^]^

**Figure 1:**
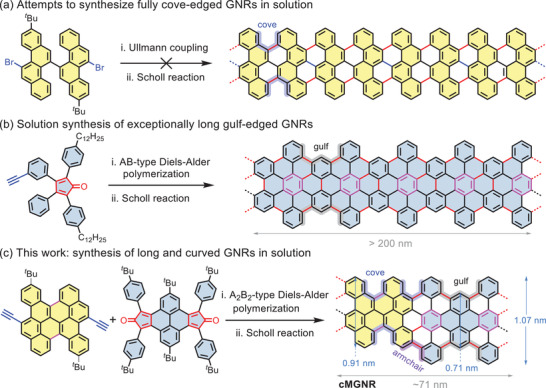
a) Attempts to synthesize fully cove‐edged graphene nanoribbons (GNRs) in solution. b) AB‐type Diels–Alder polymerization to realize exceptionally long gulf‐edged GNRs in solution. c) Synthetic route of long and curved GNRs based on A_2_B_2_‐type Diels–Alder polymerization. The functional groups in GNRs are omitted for clarity.

Early in 2015, we attempted to synthesize long and fully cove‐edged GNRs in solution from dibromo‐substituted bichrysene monomers through an Ullmann coupling reaction (**Figure** **1**a),^[^ ^7^ ^]^ but only short oligomers were obtained because the steric hindrance at the bay positions of bichrysene impedes efficient polymerization in solution. In contrast, the Diels–Alder polymerization protocol has enabled the preparation of polyphenylene precursor polymers with high molecular weight, thereby producing longer GNRs,^[^ ^8^ ^]^ with lengths surpassing these of synthesized by other polymerization methods, such as Suzuki–Miyaura, Ullmann, and Yamamoto polymerization.^[^ ^3a,d^ ^]^ For instance, we have realized the synthesis of exceptionally long GNRs (>200 nm) via AB‐type Diels–Alder polymerization from a bifunctional ethynyl‐substituted cyclopentadienone monomer (Figure 1b).^[^ ^9^ ^]^ However, AB‐type Diels–Alder polymerization for the synthesis of long GNRs is thus far limited to the above ethynyl‐substituted cyclopentadienone monomer, endowing GNRs with only a gulf‐edge topology and a rather planar conformation.^[^ ^9^, ^10^ ^]^ Under this scenario, it is desirable to design new precursors for the synthesis of long GNRs with unique edge topologies and geometries based on the Diels–Alder polymerization approach.

In this work, we demonstrated the successful synthesis of a novel, curved multi‐edged GNR (**cMGNR**) with unprecedented hybrid cove–armchair–gulf edges via the A_2_B_2_‐type Diels–Alder polymerization from the newly designed bisdienophile monomer based on a diethynyl‐substituted prefused bichrysene (**3b**) and functionalized dicyclopenta[*e*,*l*]pyrene‐5,11‐dione monomer (**6**), followed by the Scholl reaction (Figure 1c). As the “cutouts” from the **cMGNR**, compounds **1a** and **1b** were synthesized as model compounds to prove the feasibility of the Scholl reaction of bichrysene‐based precursor. Density functional theory (DFT) simulations reveal that the **cMGNR** adapts a unique curved conformation mostly due to the presence of abundant cove‐edge structures, and its lateral width lies within the range of 0.71‐1.07 nm (Figure 1c). The resultant **cMGNR** has an average length of ≈71 nm, as estimated by GPC analysis of the corresponding precursor polymer (**P1**), which is obviously longer than other reported curved GNRs.^[^ ^5^, ^7^, ^11^ ^]^ The chemical structure and optical properties of **cMGNR** were characterized by means of solid‐state NMR, FTIR, Raman, and UV–vis spectroscopic analyses. An optical bandgap of 1.61 eV is estimated for **cMGNR**, which is in line with the calculated value (1.56 eV). Moreover, time‐resolved terahertz spectroscopy reveals a charge‐carrier mobility of ≈2 cm^2^ V^–1^ s^–1^ in the **cMGNR**. Our study presents an efficient synthesis strategy to achieve a low‐bandgap, long, and curved GNR with unique hybrid edge structures, providing a promising candidate for the fabrication of single GNR‐based nanoelectronic devices.

## 2 Results and Discussion

### 2.1 Synthesis and Characterization of the Model Compounds

**Scheme 1:**
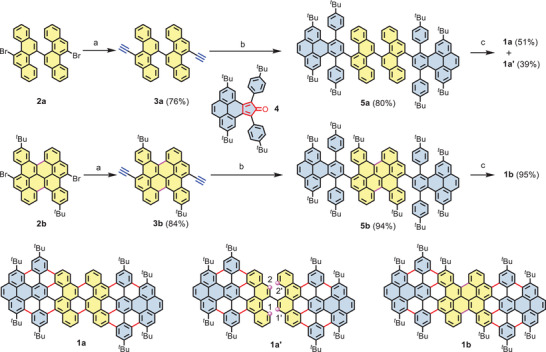
Synthetic route toward model compounds **1a** and **1b**. Reagents and conditions: a) i) PdCl_2_(PPh_3_)_2_, CuI, TIPS‐acetylene, tetrahydrofuran (THF), Et_3_N, 70 °C, 12 h; ii) tetrabutylammonium fluoride , THF, 0 °C, 15 min. b) *o*‐xylene, **4**, 170 °C, 10 h. c) FeCl_3_ (7 equiv./H), CH_3_NO_2_, CH_2_Cl_2_, rt, 30 h for **5a**; 2 h for **5b**.

To estimate the efficiency of the Scholl reaction for such particular geometry of precursor polymer for the targeted **cMGNR**, model compound **1a** was first designed and synthesized from bichrysene‐based oligophenylene **5a**. As depicted in **Scheme** **1**, the synthesis of 11,11“‐dibromo‐5,5”‐bichrysene (**2a**) was first performed according to our previously reported procedure.^[^ ^7^ ^]^ Then, the Sonogashira coupling of **2a** with the commercially available triisopropylsilylacetylene followed by treatment with tetrabutylammonium fluoride provided 11,11“‐diethynyl‐5,5”‐bichrysene (**3a**) with a yield of 76% in two steps. Moreover, 2,7‐di‐*tert*‐butyl‐9,11‐bis(4‐(*tert*‐butyl)phenyl)‐10*H*‐cyclopenta[*e*]pyren‐10‐one (**4**) was prepared. Afterward, the key precursor **5a** containing the bichrysene unit was achieved by a Diels–Alder cycloaddition reaction between **3a** and **4** in *o*‐xylene at 170 °C for 10 h in 80% yield. With precursor **5a** in hand, the Scholl reaction toward **1a** was examined. As monitored by matrix‐assisted laser desorption/ionization‐time‐of‐flight mass spectrometry (MALDI‐TOF MS), the reaction always gave a mixture of the desired product (**1a**) and the partially cyclized byproduct (**1a’**), even with an extended reaction time (30 h) and a large amount of FeCl_3_ (7 equiv./H) (Figures S1 and S2, Supporting Information). Interestingly, byproduct **1a'** could be efficiently separated by silica‐based chromatography with a yield of 39%. The structure of **1a'** was confirmed by high‐resolution MALDI‐TOF MS (Figure S4, Supporting Information) and NMR spectroscopy (Figures S6–S9, Supporting Information), in which the two C−C bond formations in the central bichrysene unit did not occur (pink dots in the structure of **1a'** in Scheme 1). This result could be rationalized by the highly distorted structure of the partially cyclized byproduct **1a'**. The carbon atoms (1/1**'** and 2/2**'**) on the bichrysene units of **1a'** (pink dots in the structure of **1a'**, Scheme 1) are distantly separated (3.28 Å) between the two blades (Figure S1, Supporting Information), which makes further dehydrogenations difficult. Therefore, target model compound **1a** was obtained in 51% yield, which was also confirmed by high‐resolution MALDI‐TOF MS analysis (Figure S3, Supporting Information). Due to its strong aggregation in common organic solvents (i.e., C_2_D_2_Cl_4_, toluene‐d_8_), it is not possible to record its ^1^H NMR spectrum, even at high temperature (120 °C). Based on the above results, we adopted a new strategy that employs precursor **5b** by prefusing the bichrysene moiety at the early stage to improve the efficiency of the Scholl reaction.

**Figure 2:**
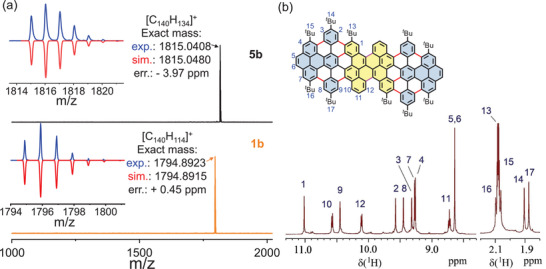
a) High‐resolution matrix‐assisted laser desorption/ionization‐time‐of‐flight mass (MALDI‐TOF) mass spectra of **5b** and **1b**. b) ^1^H NMR spectrum of **1b** in toluene‐d_8_ (500 MHz, 30 °C).

Based on a similar synthetic strategy as that for **5a**, the key precursor **5b** was synthesized starting from 9,18‐dibromo‐3,12‐di‐*tert*‐butylbenzo[*a*]dinaphtho[2,1,8‐*cde*:1’’2’’,3’’,4’’‐*ghi*]perylene (**2b**). Compared to precursor **5a**, the central bichrysene unit in **5b** was fully fused, leaving only the outer diphenylbenzo[*e*]pyrene units for the subsequent Scholl reaction. To our delight, the final cyclodehydrogenation of **5b** using FeCl_3_ (7 equiv./H) proceeded smoothly at room temperature, yielding the desired product **1b** with a high yield of 95% within only 2 h of reaction time. The efficient formation of model compound **1b** from **5b** was first validated by MALDI‐TOF analysis (**Figure** **2**a). In positive mode, the intense peak at *m/z* = 1815.0408 of **5b** disappeared, and a new signal at 1794.8923 was clearly obtained, precisely matching the calculated molecular mass [*M* ^+^] = 1794.8915 for C_140_H_114_ and the simulated isotopic distribution pattern. Thanks to the additional two *tert*‐butyl groups (position 13 in **1b**) on the armchair edges, compound **1b** becomes more soluble than its analog **1a**. The chemical structure of **1b** was unambiguously confirmed by ^1^H NMR spectroscopy (Figure 2b) with the help of 2D NMR measurements (Figures S10–S13, Supporting Information).

### 2.2 Synthesis and Characterization of the cMGNR

**Scheme 2:**
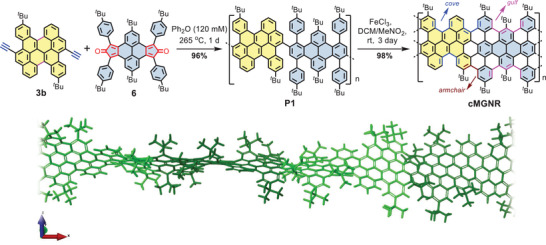
Synthetic route to the curved multi‐edged graphene nanoribbon **(cMGNR)** and density functional theory (DFT) optimized geometry of a short segment of **cMGNR**.

Encouraged by the successful synthesis of model compound **1b** from **5b**, we carried out synthesis of the **cMGNR** from the precursor polymer (**P1**) containing the prefused bichrysene unit. As shown in **Scheme** **2**, the bisdiene monomer, i.e., 2,8‐di‐*tert*‐butyl‐4,6,10,12‐tetrakis(4‐(*tert*‐butyl)phenyl)dicyclopenta[*e*,*l*]pyrene‐5,11‐dione (**6**), was first prepared in a similar manner to the synthesis of diene **4**. Then, A_2_B_2_‐type Diels–Alder polymerization was carried out by refluxing a solution of monomer **3b** and monomer **6** in diphenyl ether (120 × 10^−3^ m, 24 h) without the addition of any other reagent or catalyst, affording the polyphenylene precursor **P1** in 96% yield. Three polymer fractions were obtained through recycling preparative gel permeation chromatography (rGPC). The number‐average molecular weight (*M*_n_) of the main fraction of **P1** (75 wt%) was estimated to be ≈57 000 g mol ^−1^ with a polydispersity index (PDI) of 1.9 based on analytical GPC analysis against polystyrene (PS) standards (Figure S16, Supporting Information). Moreover, linear‐mode MALDI‐TOF MS analysis of **P1** showed periodic pattern peaks up to *m/z* ≈25 000 with an interval of *m*/*z* = 1502, corresponding to the mass of one repeating unit (Figure S17, Supporting Information). The length characterization of **cMGNR** through the scanning tunneling microscopy /atomic force microscope techniques was not successful so far, due to its highly twisted geometry and weak affinity on highly oriented pyrolytic graphite . Finally, the targeted **cMGNR** with a unique curved conformation and hybrid cove–armchair–gulf edges (Scheme 2) was obtained from **P1** through the optimized Scholl reaction with FeCl_3_ (7 equiv. per H to be removed) in CH_2_Cl_2_ for 3 days.

**Figure 3:**
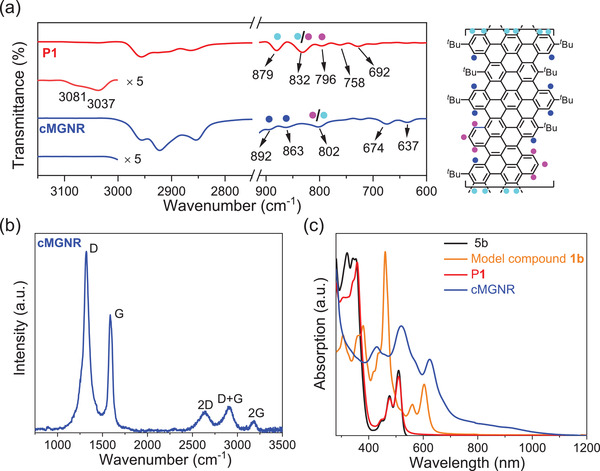
a) FTIR spectra of **P1** and the **cMGNR** measured on powder samples. b) Raman spectrum of the **cMGNR** recorded at 514 nm. c) UV‐vis‐NIR absorption spectra of **5b, 1b,** and **P1** (10^−5^ m) as well as the **cMGNR** (0.1 mg mL^−1^) in THF.

The chemical structure of **cMGNR** was then fully characterized by a combination of FTIR, Raman, and solid‐state NMR analyses. FTIR analysis of the **cMGNR** shows that, compared to polymer **P1**, the weak signals from the typical aromatic C−H stretching vibrations (broad peaks at 3037 and 3081 cm^−1^ for **P1**) were diminished, indicating efficient “graphitization” (**Figure** **3**a), which matched well with the simulation (Figure S28, Supporting Information).^[^ ^12^ ^]^ Furthermore, the most relevant fingerprint between **P1** and **cMGNR** is the SOLO mode (wagging of an isolated aromatic C−H bond neighbored by two C−C bonds). The **cMGNR** is characterized by the SOLO mode of new peaks at 863 and 892 cm^−1^ (blue in Figure 3a) of **P1**, while both peaks are absent in the spectrum of **P1**.^[^ ^12^ ^]^ We found that the intensity of the DUO mode (wagging of two adjacent aromatic C−H bonds) at 832 and 879 cm^−1^ (sky blue in Figure 3a) significantly decreased, which indicates that the DUO mode arises only from the terminals of **cMGNR** (802 cm^−1^). The peak at approximately 800 cm^−1^, remains consistent in the spectra of both the **cMGNR** and **P1**, which represents the TRIO mode (wagging of triply adjacent C−H groups, pink in Figure 3a).^[^ ^12^ ^]^ Notably, the peaks at 758, 692, 674, and 637 cm^−1^ are assigned to the out‐of‐plane and in‐plane bending modes of the carbon atoms of **P1** and the **cMGNR**. In the Raman spectrum of **cMGNR**, two intense peaks at approximately 1322 and 1588 cm^−1^ were observed (Figure 3b), which can be assigned to the D and G bands of graphitic materials, respectively. This result is consistent with the literature values for the reported bottom‐up solution‐synthesized GNRs.^[^ ^13^ ^]^ The relatively high intensity of the D band can be explained by the contribution of the edges as defects. Moreover, three well‐resolved double‐resonance signals were also observed at 2644, 2911, and 3179 cm^−1^, which can be assigned to 2D, D + G, and 2G peaks, respectively. Solid‐state ^1^H and ^13^C{^1^H} MAS NMR experiments of **P1** and **cMGNR** (Figures S18 and S19, Supporting Information) validate the successful formation of the **cMGNR** as obtained from quantitative analysis of the aromatic and aliphatic signals (Table S1, Supporting Information). 2D ^1^H‐^1^H DQ‐SQ NMR correlation experiments (Figure S18, Supporting Information) further reveal a reduced span of the aromatic ^1^H‐^1^H autocorrelation signal (up to ≈11 ppm) for **cMGNR**, which is associated with the reduced *π*–*π* stacking interactions in comparison to those of other GNR samples (up to ≈15 ppm).^[^ ^3^, ^9^, ^10^, ^14^ ^]^ This observation further supports the nonplanar geometry of the **cMGNR** as depicted in Scheme 2.

The UV–vis absorption spectra of model compound **1b** and the **cMGNR**, as well as their precursors (**5b** and **P1**), were recorded in tetrahydrofuran solutions (Figure 3c). In comparison to those of precursors **5b** and **P1**, the absorption peaks of **1b** and the **cMGNR** display a significant redshift, due to their *π*‐extended conjugation after the cyclodehydrogenation reactions. The longest absorption peak appears at 605 nm for **1b**, which is assignable to the HOMO to LUMO electronic transition according to time‐dependent DFT calculations (Table S3, Supporting Information). The optical bandgap of **1b** is calculated to be 1.81 eV, based on the Tauc plot of UV–vis absorption, which is in good agreement with the calculated result (1.87 eV, Scheme S22, Supporting Information). Remarkably, the **cMGNR** exhibits a strong, broad absorption covering the UV, visible, and even near‐infrared (NIR) region, indicating the efficient “graphitization” of polymer precursor **P1**. From the onset of its longest absorption peak at 621 nm, the optical bandgap of **cMGNR** is calculated to be 1.61 eV, in excellent agreement with the calculated result (1.56 eV, Figure S23, Supporting Information).

### 2.3 THz Study of the cMGNR

**Figure 4:**
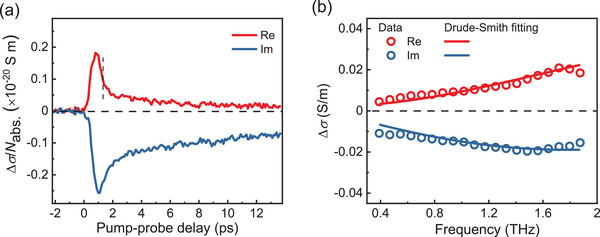
a) Time‐resolved complex terahertz photoconductivity of the **cMGNR** dispersed in 1,2,4‐trichlorobenzene, normalized to the absorbed photon density. b) Frequency‐resolved terahertz conductivity measured at ≈1.3 ps after photoexcitation (denoted by the dashed vertical line in (a)). The solid lines are fits using the Drude–Smith model.

To study the photoconductivity dynamics and transport properties of charge carriers in **cMGNR**, we employed contact‐free optical pump‐terahertz (THz) probe (OPTP) spectroscopy. **Figure** **4**a presents the complex photoconductivity dynamics of the **cMGNR** dispersed in 1,2,4‐trichlorobenzene. An initial rise in photoconductivity is observed due to the transient population of free carriers following interband excitations (by a laser pulse with a photon energy of 3.10 eV). Subsequently, free carriers are transformed into exciton species, resulting in a rapid ≈ps decay in the real conductivity and a long‐lived imaginary contribution to the photoconductivity.^[^ ^5^, ^9^, ^15^ ^]^ To further study the free charge carrier transport properties at an early timescale, we measured the frequency‐resolved complex conductivity spectrum (at ≈1.3 ps after photoexcitation), and described the data using the Drude–Smith (DS) model (Figure 4b; see Supporting Information for more details).^[^ ^16^ ^]^ In the model, the charge carriers are assumed to be subject to anisotropic momentum scattering processes, particularly with an increasing preferential backscattering probability, due to the presence of, e.g., grain boundaries and torsional defects in the materials.^[^ ^5b^ ^]^ A parameter *c* is introduced to characterize the backscattering probability, which ranges between 0 (isotropic scattering) and −1 (fully backscattering). DS model analysis (solid lines in Figure 4b) yields the charge scattering time *τ* = 36 ± 2 fs and *c* = −0.97 ± 0.01. Furthermore, knowing the effective mass of the charge carriers (m*_h_ = 2.16 *m*_0_ and m*_e_ = 1.27 *m*_0_, see details in Supporting Information), we infer the mobility of the charge carriers in the dc limit to be 2.4 cm^2^ V^−1^ s^−1^, following *μ* =$\frac{e\tau }{m^{*}}\;\left(1+c\right)$. The obtained mobility of the **cMGNR** is relatively lower than other reported curved GNRs,^[^ ^5d,e^ ^]^ mostly due to its large effective mass and flat band dispersion (Figure S27, Supporting Information).

## 3 Conclusion

In summary, we have demonstrated an efficient strategy to synthesize a novel long and curved GNR with combined cove, armchair, and gulf edge structures, namely, **cMGNR**. The synthetic route of the **cMGNR** involves efficient A_2_B_2_‐type Diels–Alder polymerization by using a diethynyl‐substituted fused bichrysene derivative (**3b**) as a bisdienophile monomer and dicyclopenta[*e*,*l*]pyrene‐5,11‐dione (**6**) as a bisdiene monomer, followed by intramolecular cyclodehydrogenation. Model compound **1b** was efficiently synthesized from prefused bichrysene‐based oligomer **5b**, which demonstrates the suitable structural design of precursor polymer **P1**. Remarkably, the obtained **cMGNR** had an average length of ≈71 nm and a low optical bandgap of 1.61 eV. In addition, THz photoconductivity analysis of the **cMGNR** revealed a charge carrier mobility exceeding 2 cm^2^ V^−1^ s^−1^, which makes it a promising candidate for single‐GNR based nanoelectronic devices. This work opens a door for the synthesis of novel GNRs with high longitudinal extension and nonplanar conformation via A_2_B_2_‐type Diels–Alder polymerization by using other bisdiene and bisdienophile monomers.

## Conflict of Interest

The authors declare no conflict of interest.

## Supporting information

Supporting Information

## Data Availability

The data that support the findings of this study are available from the corresponding author upon reasonable request.
