# Supplementary material for: Solution Synthesis and Characterization of a Long and Curved Graphene Nanoribbon with Hybrid Cove–Armchair–Gulf Edge Structures
Source: Adv Sci (Weinh). 2022 Mar 24;9(19):2200708. doi: 10.1002/advs.202200708 (PMC9259722; doi:10.1002/advs.202200708)
Supplement: Supplementary file 1 — Supporting Information [file ADVS-9-2200708-s001.pdf]

ADVANCED SCIENCE
Open Access

Supporting Information

for *Adv. Sci.*, DOI 10.1002/advs.202200708

Solution Synthesis and Characterization of a Long and Curved Graphene Nanoribbon with Hybrid Cove–Armchair–Gulf Edge Structures

Lin Yang, Ji Ma, Wenhao Zheng, Silvio Osella, Jörn Droste, Hartmut Komber, Kun Liu, Steffen Böckmann, David Beljonne, Michael Ryan Hansen, Mischa Bonn, Hai I. Wang, Junzhi Liu and Xinliang Feng**

Supporting Information

Solution Synthesis and Characterization of a Long and Curved Graphene Nanoribbon with Hybrid Cove-Armchair-Gulf Edge Structures

Lin Yang, Ji Ma, Wenhao Zheng, Silvio Osella, Jörn Droste, Hartmut Komber, Kun Liu, Steffen Böckmann, David Beljonne, Michael Ryan Hansen, Mischa Bonn, Hai I. Wang, Junzhi Liu, Xinliang Feng**

Table of contents

1. General methods and materials S3

2. Detailed synthetic procedure and characterization data of **1a** and **1b** S5

3. Detailed synthetic procedure and characterization data of **P1** and **the cMGNR** S20

4. Solid-state NMR characterization of **P1** and **the cMGNR** S24

5. DFT calculations S28

6. Terahertz spectroscopic study of the **cMGNR** S38

7. NMR spectra S39

8. High-resolution mass spectroscopy (HR-MS) S49

9. References S51

1. General methods and materials

All the reagents were obtained from Sigma Aldrich, TCI, abcr, Alfa Aesar, Strem, fluorochem, and chempur. All these chemicals were used as received without further purification. All reactions dealing with air- or moisture-sensitive compounds were carried out in a dry reaction vessel under argon (Ar) atmosphere by using standard vacuum-line and Schlenk techniques. Anhydrous dichloromethane and tetrahydrofuran were obtained from MBRAUN MB-SPS-5 solvent purification system.

Thin layer chromatography (TLC) was performed on silica-coated aluminium sheets with a fluorescence indicator (TLC silica gel 60 F254, purchased from Merck KGaA).

Column chromatography was performed on silica (SiO_2, particle size 0.063-0.200 mm, purchased from VWR).

NMR spectra were recorded on a Bruker Avance III 500 spectrometer operating at 500.13 MHz for ^{1}H and at 125.77 MHz for ^{13}C at 30°C (unless otherwise stated). The 1D and 2D NMR spectra were recorded using the standard Bruker pulse programs. CD_2Cl_2 (δ(^{1}H) = 5.33 ppm, δ(^{13}C) = 53.7 ppm), $C_2D_2Cl_4$ (δ(^{1}H) = 5.98 ppm, δ(^{13}C) = 73.7 ppm) and toluene-d_8 (δ(^{1}H) = 2.08 ppm, δ(^{13}C) = 20.4 ppm) were used as solvents and as internal chemical shift reference. Chemical shifts (δ) are reported in ppm. The following abbreviations are used to describe peak patterns as appropriate: s = singlet, d = doublet, t = triplet, q = quartet, and m = multiplet.

Relative molar masses were determined by gel permeation chromatography (GPC) with an Aligent Technologies 1260 Infinity LC system equipped with two Resipore columns and RI and UV–vis detection. Chloroform was used as eluent with a flow rate of 1 mL min^{-1}. The measurements were carried out at 40 ºC. The molar masses were calculated relative to polystyrene standards with low dispersity.

High-resolution ESI mass spectra were recorded in the positive mode with a Finnigan LTQ-FT from Fisher Thermo Scientific.

The high-resolution matrix-assisted laser desorption/ionization time-of-flight (MALDI-TOF) mass spectrometry was performed on a Bruker Autoflex Speed MALDI TOF MS (Bruker Daltonics, Bremen, Germany) using *trans*-2-[3-(4-*tert*-butylphenyl)-2-methyl-2-propenylidene]malononitrile (DCTB) as matrix.

UV–visible spectra were measured on an Agilent Cary 5000 UV–vis–NIR spectrophotometer by using 10 mm optical-path quartz cell at room temperature.

Fluorescence spectra were recorded at room temperature on a Perkin-Elmer Fluorescence Spectrometer LS 55 using a 10 mm fluorescence quartz cell.

Raman spectroscopy was conducted using a confocal Raman microscope (S&I Monovista CRS +) with laser excitation by a Toptica Top mode single frequency 514 nm diode laser.

The solid-state NMR experiments were conducted on a Bruker AVANCE NEO spectrometer (11.76 T, $\nu_L(^1H)$ = 500.39 MHz) using a Bruker 1.3 mm H/F/X/Y MAS DVT probe. The samples were packed into 1.3 mm o.d. ZrO_2 rotors and sealed with Vespel© top and bottom caps. Adamantane was used as external reference to determine the radio-frequency (rf) pulse length ($t_{\pi/2}(^1H)$ = 1.9 μs, $t_{\pi/2}(^{13}C)$ = 5.0 μs) and to calibrate the chemical shift scale ($\delta(^1H)$ = 1.85 ppm, $\delta(^{13}C)$ = 29.47 ppm).[1] The $^{13}C\{^1H\}$ MAS NMR experiments were recorded with low power ^1H cw (ν_{rf} (^1H) = 30 kHz) decoupling. A spin echo with an echo time of two rotor periods (τ_{echo}= $2\tau_r$=40 or 32 μs) was applied to remove the probe background signals in the ^1H NMR spectra. For the ^1H MAS NMR spectra a recycle delay of 16 s and 4 s proved sufficient for **P1** and **cMGNR**, respectively. For the $^{13}C\{^1H\}$ MAS NMR experiments a recycle delay of 3.5 s and 22 s was applied. It was shown previously that a recycle delay of 3.5 s is sufficient for GNR samples.[2] However, with this short recycle delay, no quantitative analysis for **P1** could be performed and in this study, the recycle delay for **cMGNR** had to be increased to obtain close to quantitative information. In interest of time, however, no longer recycle delay for **P1** could be utilized. The 2D ^1H-^1H DQ-SQ NMR correlation spectra were recorded with eight rotor periods of DQ recoupling/reconversion using the BaBaXY16 phase cycle scheme.[3] Data processing and analysis was done using the Bruker Topspin4.0.9 software, dmfit2019,[4] and Python.

2. Detailed synthetic procedure and characterization data of 1a and 1b

2.1 Synthesis of model compounds 1a and 1b

11,11'-Dibromo-5,5'-bichrysene (**2a**),[5] 9,18-dibromo-3,12-di-*tert*-butylbenzo[*a*]dinaphtho[2,1,8-*cde*:1',2',3',4'-*ghi*]perylene (**2b**),[6] 2,7-di-*tert*-butyl-9,11-bis(4-(*tert*-butyl)phenyl)-10*H*-cyclopenta[*e*]pyren-10-one (**4**)[7] and 2,8-di-*tert*-butyl-4,6,10,12-tetrakis(4-(*tert*-butyl)phenyl)dicyclopenta[*e*,*l*]pyrene-5,11-dione (**6**)[7] were prepared according to literature methods.

11,11'-Diethynyl-5,5'-bichrysene (3a)

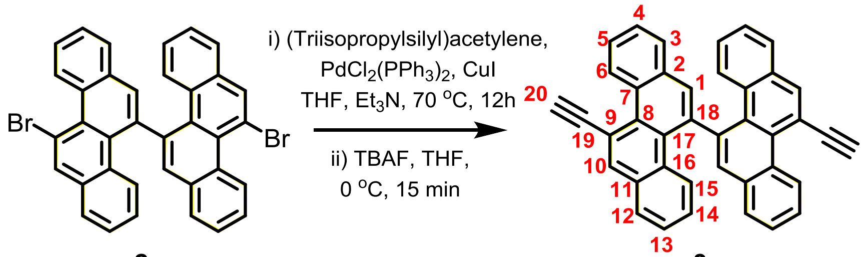

A mixture of compound **2a** (300 mg, 0.49 mmol), $PdCl_2(PPh_3)_2$ (34.1 mg, 0.049 mmol), CuI (18.5 mg, 0.098 mmol), Et_3N (3 mL) and THF (30 mL) in a 50 mL Schlenk flask was purged with Ar for 30 min. Then, degassed triisopropylsilylacetylene (0.34 mL, 1.5 mmol) was added via syringe. The reaction mixture was stirred at 50 °C for 12 h. After being cooled to room temperature, the solvent was removed under reduced pressure, and the residue was subjected to flash chromatography (silica gel, isohexane/CH_2Cl_2=3/1) and used for desilylating directly. Ar was bubbled through the obtained mixture in 30 mL THF for 30 min, and the 1 mL TBAF (1 M in the THF, 1 mmol) in the solution was added via dropwise at 0 °C. After 15 min (TLC monitored), the reaction was quenched with methanol. The solvent was removed under vacuum. The residue was cleaned by column chromatography (silica gel, isohexane/CH_2Cl_2=6/1) to afford the product **3a** (187.1 mg, 76%, yellow solid).

3a: ^{1}H NMR (500 MHz, CD_2Cl_2): 10.29 (d, 8.7 Hz, 2H; 6), 8.45 (s, 2H; 10), 8.12 (d, 8.7 Hz, 2H; 15), 7.87 (d, 8.0 Hz, 2H; 12), 7.73 (d, 7.9 Hz, 2H; 3), 7.70 (t, 8.0 Hz, 2H; 5), 7.68 (s, 2H; 1), 7.61 (t, 7.6 Hz, 2H; 4), 7.35 (t, 7.5 Hz, 2H; 13), 6.88 (7, 7.9 Hz, 2H; 14), 3.75 (s, 2H; 20). ^{13}C NMR (125 MHz, CD_2Cl_2): 140.7 (18), 138.3 (10), 132.5 (2), 132.1 (11), 131.5 (1), 130.5 (16), 130.1 (7, 8), 129.3 (17), 128.2 (15), 128.1 (3), 127.9 (12), 127.4 (4), 127.2 (6), 126.6 (13), 126.4 (14), 125.9 (5), 116.9 (9), 86.6 (19), 82.6 (20). HR-MS (ESI): calculated for $C_{40}H_{22}$, 503.1722; found, 502.1718, error = -0.68 ppm.

3,12-Di-*tert*-butyl-9,18-diethynylbenzo[*a*]dinaphtho[2,1,8-*cde*:1',2',3',4'-*ghi*]perylene (3b)

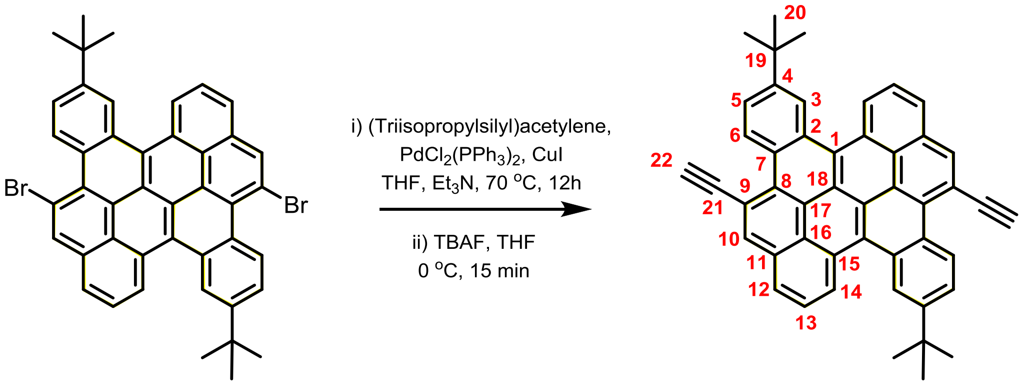

2b **3b**

Following a procedure similar to that for compound **3a**, compound **3b** was obtained as a red solid from **2b** in 84% yield.

3b: ^{1}H NMR (500 MHz, CD_2Cl_2): 10.39 (d, 9.1 Hz, 2H; 6), 9.23 (d, 2.0 Hz, 2H; 3), 8.97 (d, 7.8 Hz, 2H; 14), 8.68 (s, 2H; 10), 8.21 (d, 7.8 Hz, 2H; 12), 8.04 (t, 7.9 Hz, 2H; 13), 7.94 (dd, 9.0 Hz, 2.0 Hz, 2H; 5), 3.82 (s, 2H; 22), 1.54 (s, 18H; 20). ^{13}C NMR (125 MHz, CD_2Cl_2): 150.2 (4), 137.5 (10), 131.1 (15), 129.6 (11), 129.2 (14), 128.8 (2), 127.9 (7), 126.6 (6), 126.4 (13), 126.1 (12), 126.0 (8), 125.9 (16), 124.9 (17 or 18), 124.7 (1), 123.7 (5), 123.4 (3), 122.8 (17 or 18), 117.2 (9), 86.3 (21), 82.7 (22), 35.5 (19), 31.4 (20). HR-MS MALDI-TOF (m/z): calculated for $C_{48}H_{34}$ $[M]^{+}$, 610.2655; found, 610.2659, error = 0.66 ppm.

11,11'-Bis(2,7-di-*tert*-butyl-9,12-bis(4-(*tert*-butyl)phenyl)benzo[*e*]pyren-10-yl)-5,5'-bichrysene (5a)

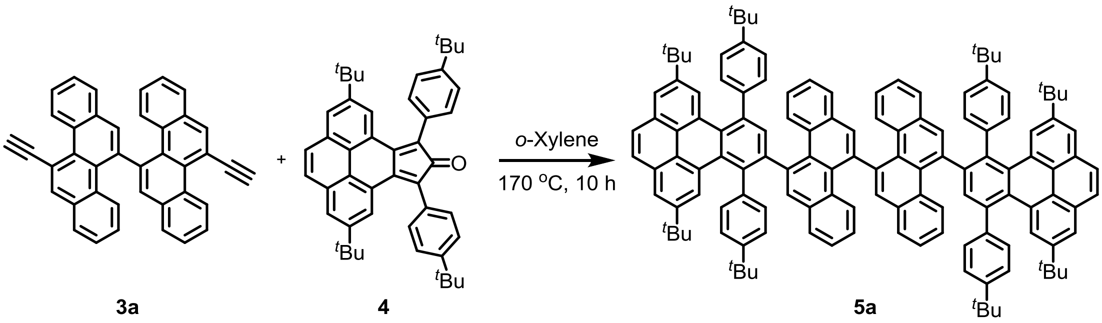

3a **4** **5a**

A degassed solution of **3a** (30.0 mg, 0.06 mmol) and **4** (79.1 mg, 0.125 mmol) in 3 mL *o*-xylene was refluxed (170 °C) for 12 h. After cooling down to room temperature, methanol was added. The precipitates were collected by filtration with a membrane filter to obtain the crude product. The residue was then purified by column chromatography (silica gel, isohexane/CH_2Cl_2=10/1), affording **5a** as orange-red solid (81.6 mg, 80%).

5a: This compound results in very complex ^{1}H and ^{13}C NMR spectra. It is very likely that different conformational isomers exist as both the variable-temperature ^{1}H NMR experiment by changing line widths and also the ROESY experiment by presence of exchange peaks indicate dynamic processes. Hindered rotation of the bulky *tert*-butyl substituted 9,12-diphenylbenzo[*e*]pyrene groups about the single bond to the core moiety should be the reason for the observed dynamic effect.

Due to the complexity of the spectra, a signal listing is omitted. The depicted ^{1}H NMR spectra is characteristic of **5a** and should provide fingerprints that can be used when comparing samples (Figure S37).

5a: HR-MS MALDI-TOF (m/z): calculated for $C_{132}H_{122}$ $[M]^{+}$, 1706.9541; found, 1706.9542, error = 0.06 ppm.

3,12-Di-*tert*-butyl-9,18-bis(2,7-di-*tert*-butyl-9,12-bis(4-(*tert*-butyl)phenyl)benzo[*e*]pyren-10-yl)benzo[*a*]dinaphtho[2,1,8-*cde*:1',2',3',4'-*ghi*]perylene (5b)

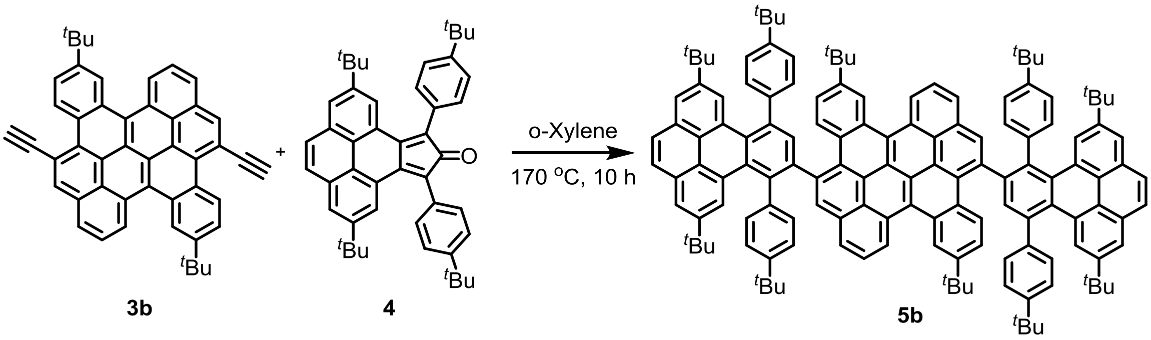

Following a procedure similar to that for compound **5a**, compound **5b** was obtained as a red solid from **3b** and **4** in 94% yield.

5b: Similar to **5a**, this compound results in very complex ^{1}H and ^{13}C NMR spectra. Due to the complexity of the spectra, a signal listing is omitted. The depicted ^{1}H, ^{13}C and 2D NMR spectra are characteristic of **5b** and should provide fingerprints that can be used when comparing samples (Figure S38-44).

5b: HR-MS MALDI-TOF (m/z): calculated for $C_{140}H_{134}$ $[M]^{+}$, 1815.0480; found, 1815.0408, error = -3.97 ppm.

Synthesis of 1a

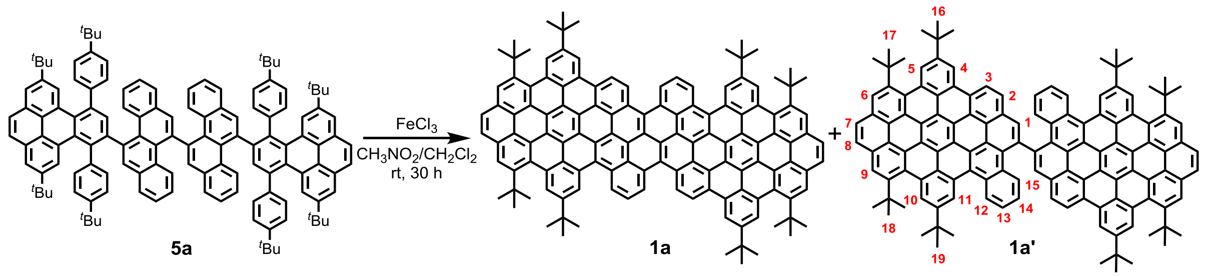

A solution of compound **5a** (30.0 mg, 17.6 μmol) in 15 mL of DCM was degassed by Ar bubbling for 15 min. To the degassed solution was added a suspension of $FeCl_3$ (478.5 mg, 2.95 mmol) in 1 mL of nitromethane. The MALDI-TOF mass spectrum indicates the mixture of partially cyclodehydrogenated intermediate (**1a′**) and the desired product (**1a**) after 15 h reaction time. The reaction time was then extended to 30 h, but MALDI-TOF mass spectrum still shows the existence of partially cyclized byproduct (**1a′**, Figure S1). After that, the reaction was stopped by addition of methanol to yield a dark red precipitate which was collected by filtration. The residue was then purified by column chromatography (silica gel, isohexane/CH_2Cl_2=5/1), affording **1a** (51%) and **1a′** (39%).

1a: Unfortunately, we could not get the resolved NMR spectra of **1a** due to its strong aggregation in solution, even at high temperature (120 ^{o}C).
HR-MS MALDI-TOF (m/z): calculated for $C_{132}H_{98}$ $[M]^+$, 1682.7663; found, 1682.7672, error = 0.53 ppm.

1a′: ^{1}H NMR (500 MHz, $C_2D_2Cl_4$, 120 °C): 9.87 (d, 8.8 Hz, 2H; 3), 9.84 (d, 8.3 Hz, 2H; 12), 9.83 (s, 2H; 11), 9.74 (s, 2H; 4), 9.42 (s, 2H; 5), 9.32 (s, 2H; 10), 9.31 (s, 2H; 9), 9.28 (s, 2H; 6), 9.23 (s, 2H; 1), 9.12 (d, 8.1 Hz, 2H; 15), 8.97 (d, 8.8 Hz, 2H; 2), 8.74 (AB system, 4H; 7 and 8), 7.72 (t, 7.8 Hz, 2H; 13), 7.00 (t, 7.8 Hz, 2H; 14), 2.13 (s, 18 H; 18), 2.04 (s, 18 H; 17), 1.97 (s, 18 H; 16), 1.95 (s, 18 H; 19).
A ^{13}C NMR spectrum could not be recorded due to the low solubility at 30°C. A long-term ^{13}C NMR measurement at 120°C was not possible for technical reasons.
HR-MS MALDI-TOF (m/z): calculated for $C_{132}H_{102}$ $[M]^+$, 1686.7976; found, 1686.7983, error = 0.41 ppm.

Synthesis of 1b

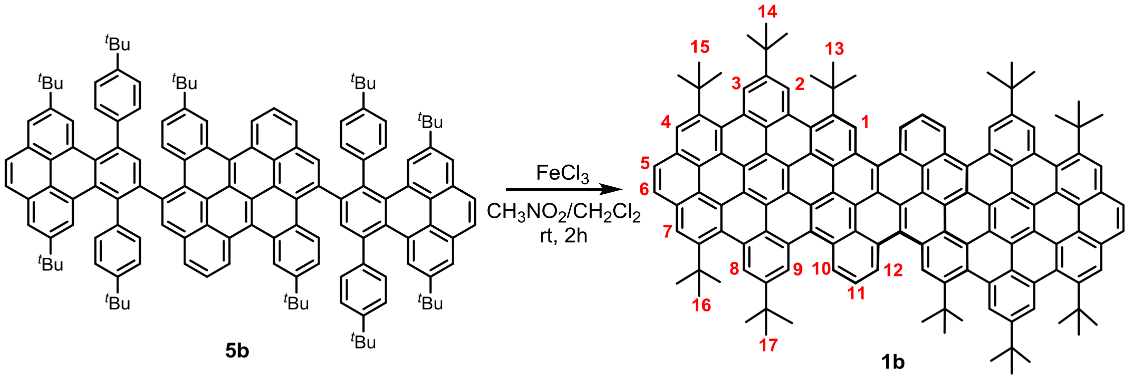

Following a procedure similar to that for compound **1a** and **1a′**, compound **1b** was obtained as a red solid from **5b** in 95% yield.

1b: This compound form aggregates in usual chlorinated NMR solvent like $CDCl_3$, CD_2Cl_2 and $C_2D_2Cl_4$ most probably due to π-π stacking of the extended planar polyaromatic system. This results in very broad signals, which does not allow a structure analysis.

Fortunately, low solubility without line broadening due to aggregation is observed in the aromatic solvent toluene-d_8. However, the solubility was not sufficient to record the ^{13}C NMR spectrum and ^{1}H-^{13}C correlated 2D NMR spectra. Therefore, only ^{1}H NMR data can be presented. Evaluation of the ^{1}H chemical shift data, the $^{3}J_{HH}$ and $^{4}J_{HH}$ coupling constants, the cross peaks in the long-range COSY spectrum and the spatial neighbourhoods derived from the ROESY spectrum confirm the presence of cove and armchair regions. In summary, the structure of **1b** is confirmed by the ^{1}H NMR data.

^{1}H NMR (500 MHz, toluene-d_8): 11.02 (s, 2H; 1), 10.57 (d, 7.9 Hz, 2H; 10), 10.45 (d, 1.5 Hz, 2H; 9), 10.11 (d, 7.6 Hz, 2H; 12), 9.56 (d, 2.0 Hz, 2H; 2), 9.44 (d, 1.5 Hz, 2H; 8), 9.31 (d, 2.0 Hz, 2H; 3), 9.26 (s, 2H; 7), 9.25 (s, 2H; 4), 8.70 (t, 7.9 Hz, 2H; 11), 8.62 (4H; 5, 6), 2.11 (s, 18H; 16), 2.09 (s, 18H; 13), 2.07 (s, 18H; 15), 1.90 (s, 18H; 14), 1.87 (s, 18H; 17).

HR-MS MALDI-TOF (m/z): calculated for $C_{140}H_{114}$ $[M]^{+}$, 1794.8915; found, 1794.8923, error = 0.45 ppm.

2.2 MALDI-TOF mass spectra of Scholl reaction for 5a and 5b

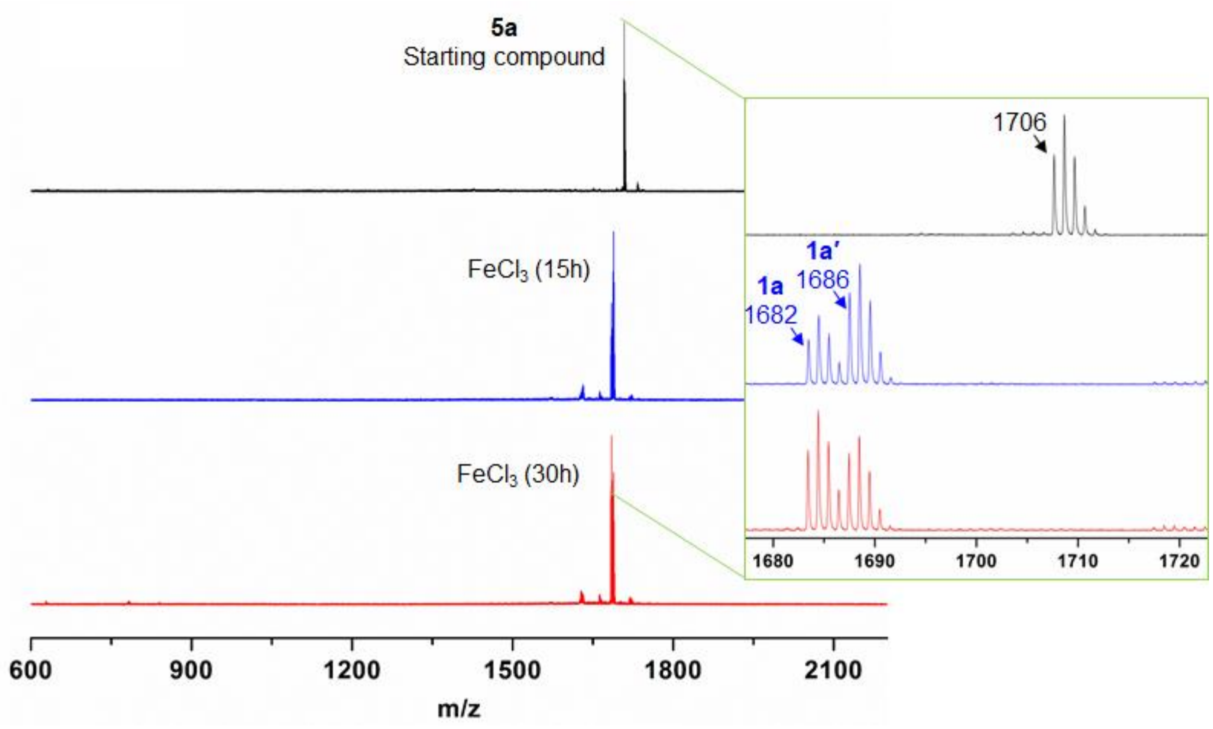

Figure S1. MALDI-TOF mass spectra of the Scholl reaction for **5a**.

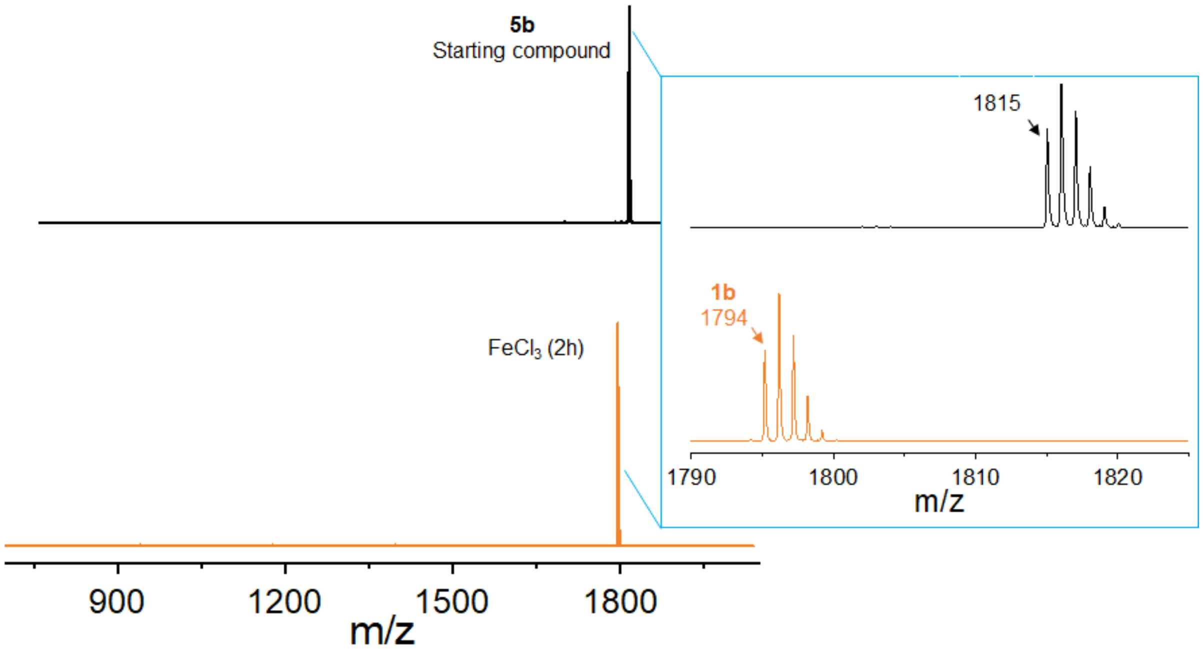

Figure S2. MALDI-TOF mass spectra of the Scholl reaction for **5b**.

2.3 HR MALDI-TOF mass spectra of 1a, 1a′ and 1b

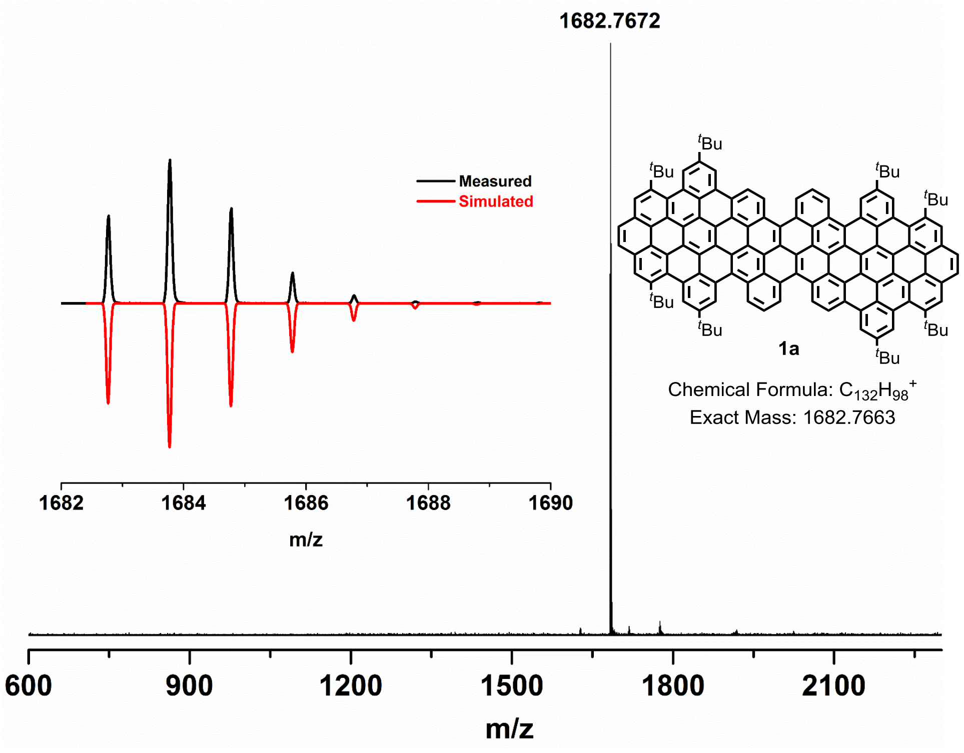

Figure S3. HR MALDI-TOF mass spectrum of **1a**.

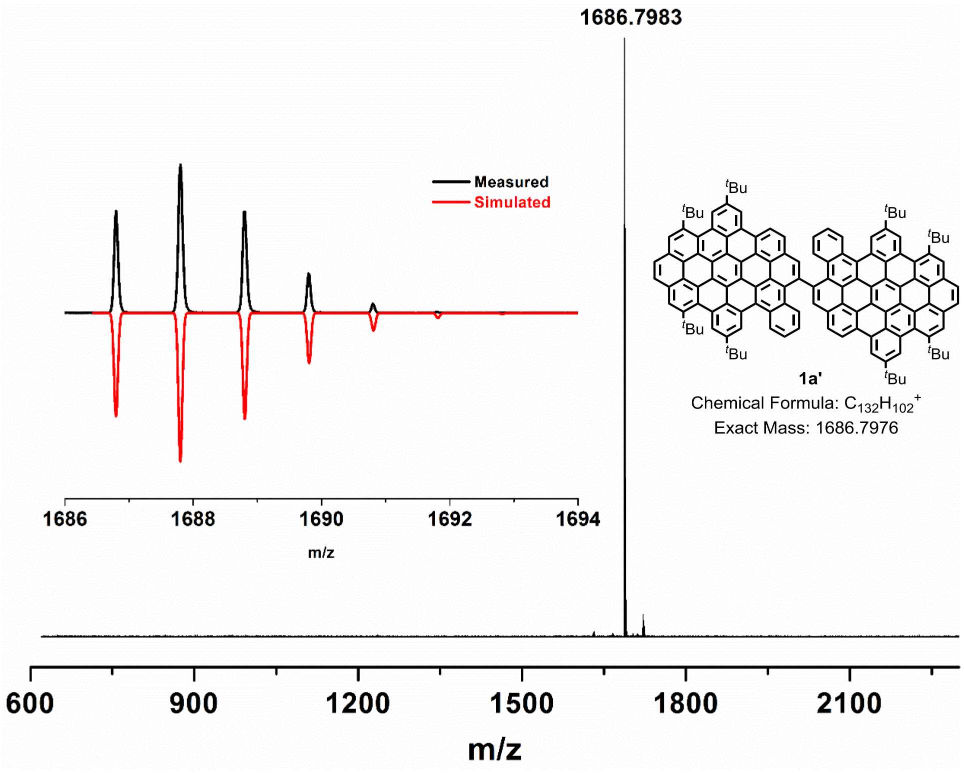

Figure S4. HR MALDI-TOF mass spectrum of **1a′**.

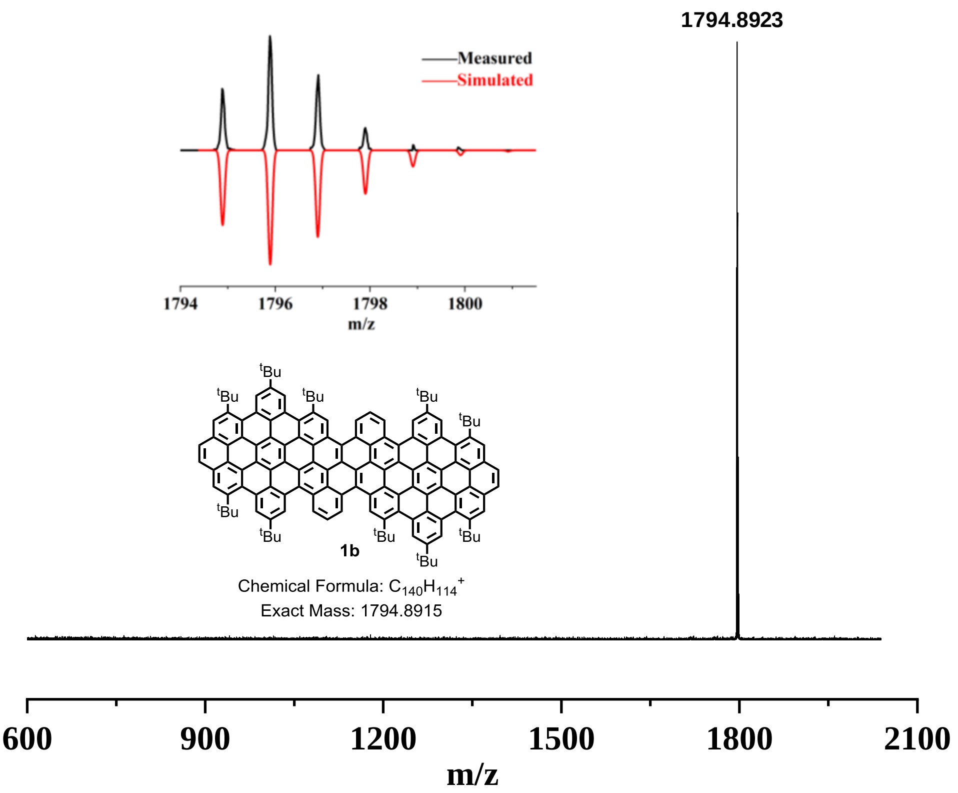

Figure S5. HR MALDI-TOF mass spectrum of **1b**.

2.4 NMR spectra of 1a′ and 1b

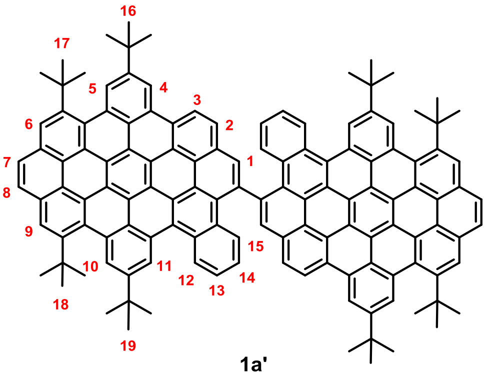

1a'

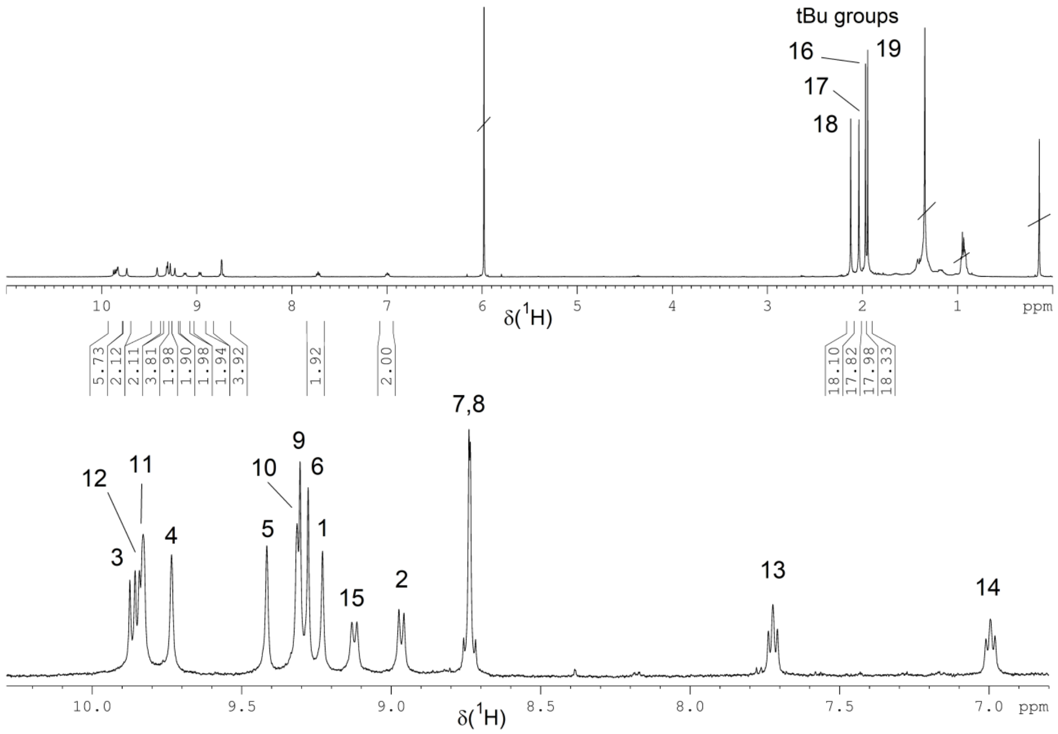

Figure S6. ^{1}H NMR spectrum of **1a′** ($C_2D_2Cl_4$, 120°C) - overview and region of aromatic protons.

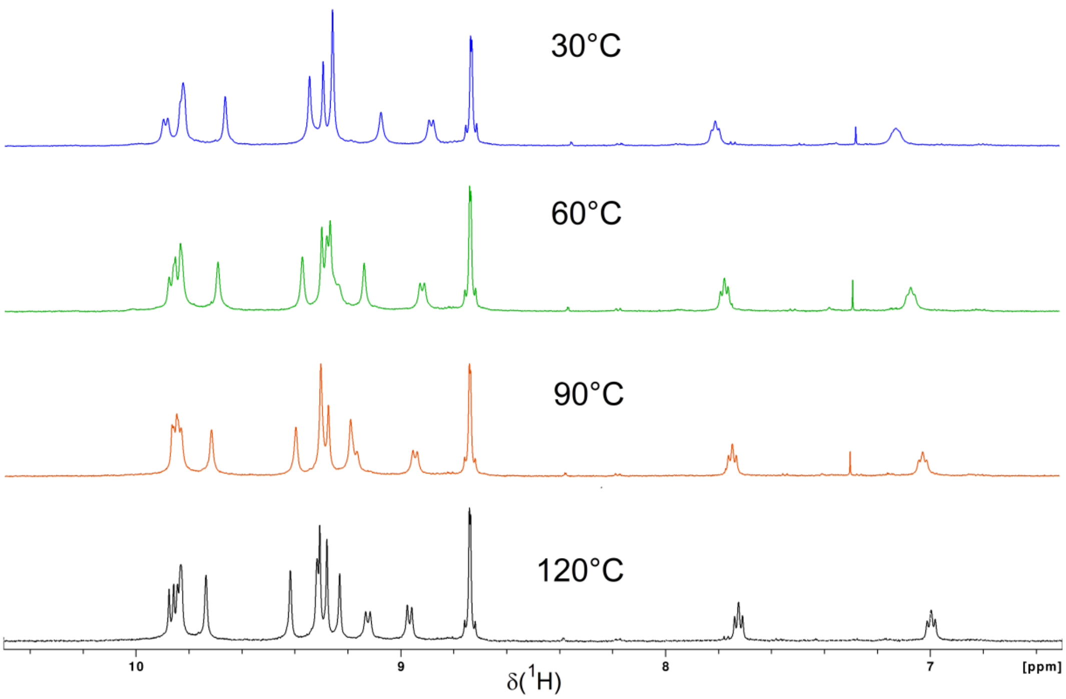

Figure S7. Variable temperature ^{1}H NMR spectra (region) of **1a′** ($C_2D_2Cl_4$).

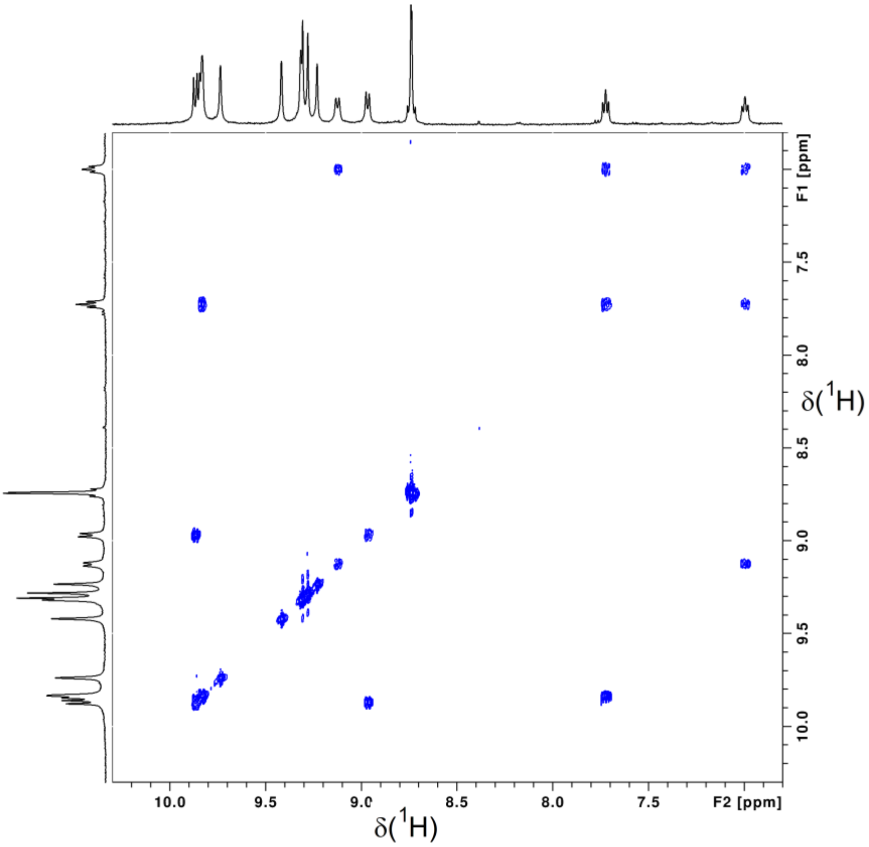

Figure S8. COSY spectrum (region) of **1a′** ($C_2D_2Cl_4$, 120°C).

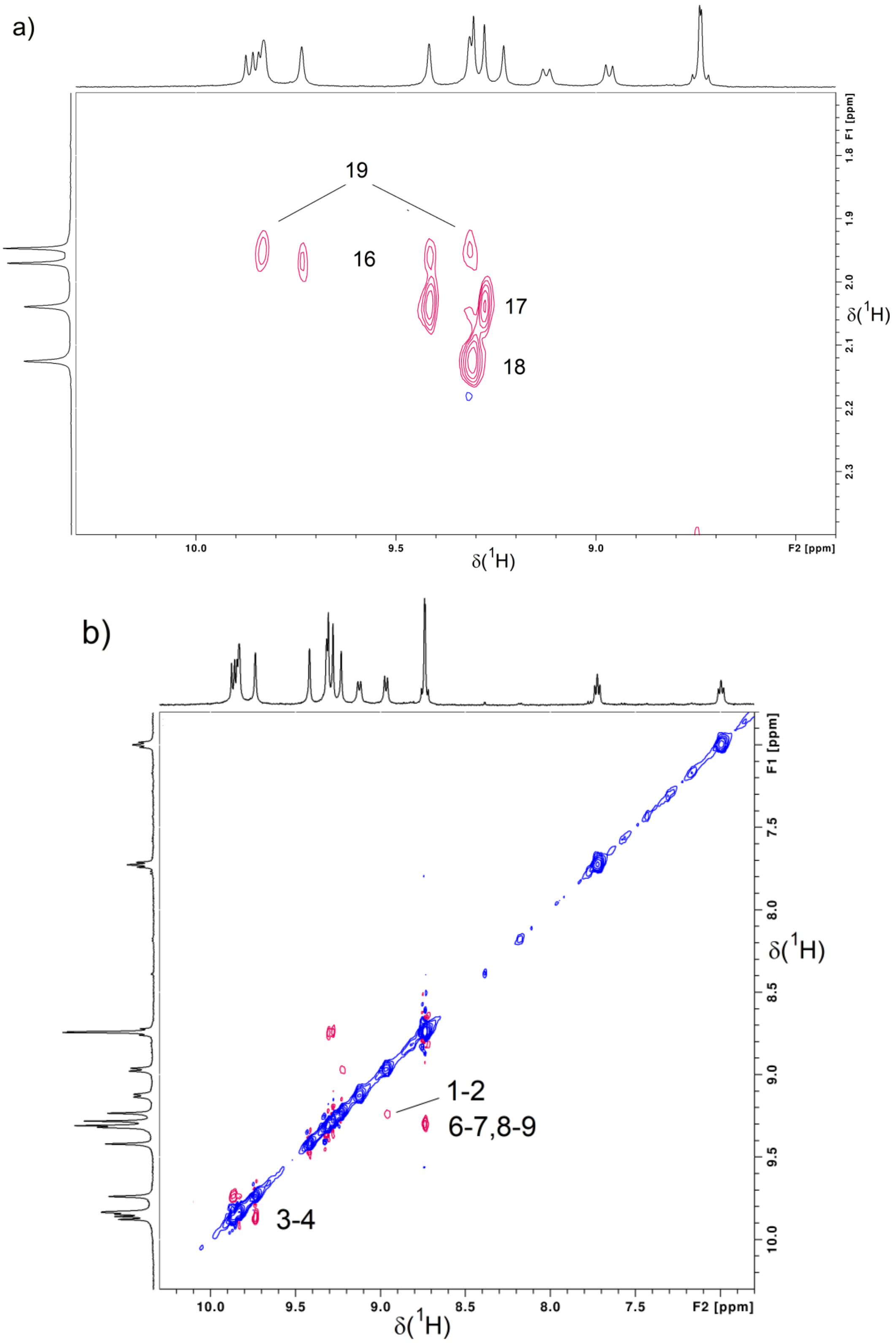

Figure S9. ROESY spectrum of **1a′** (regions) showing correlations due to spatial neighbourhoods between *tert*-butyl groups and aromatic protons (a) and between aromatic protons (b) ($C_2D_2Cl_4$, 120°C).

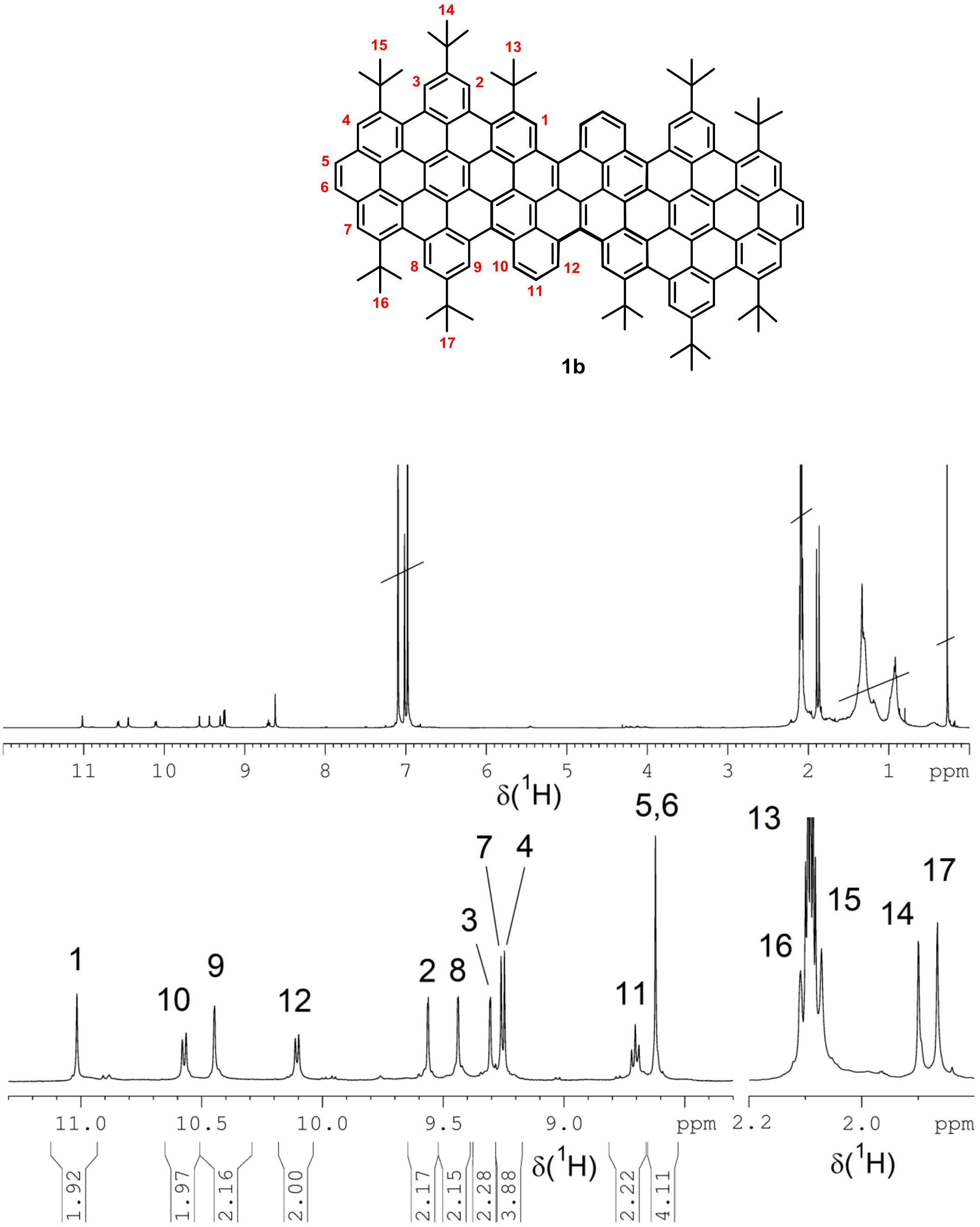

Figure S10. ¹H NMR spectrum of **1b** - overview and regions of aromatic and *tert*-butyl protons (toluene-d_8, 30°C).

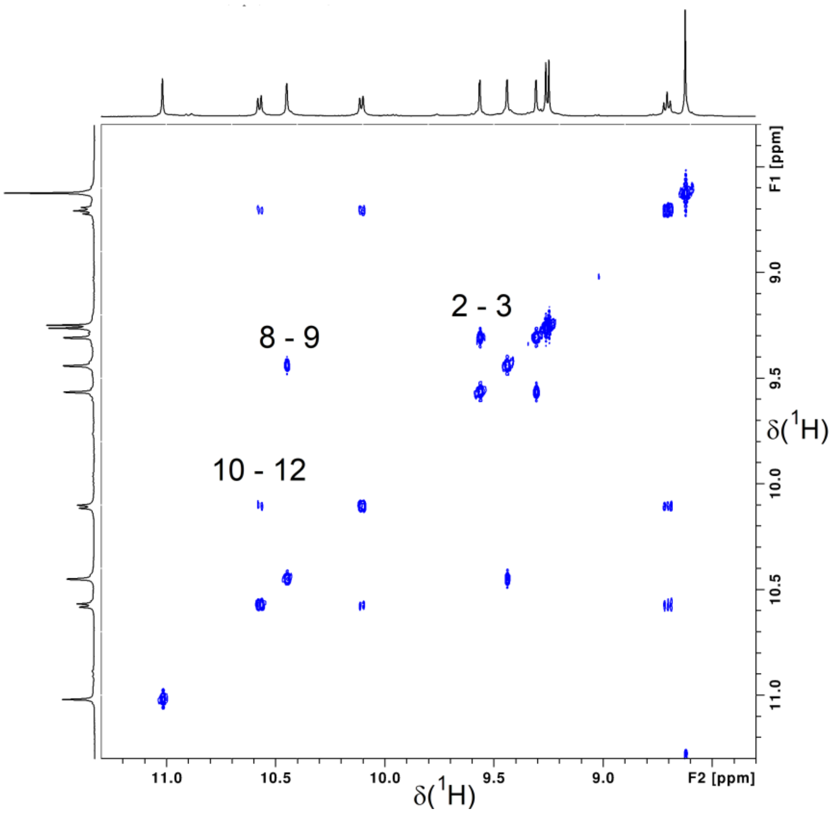

Figure S11. Long-range COSY spectrum of **1b** with assigned correlations due to ${}^{4}J_{HH}$ coupling (toluene-d_8).

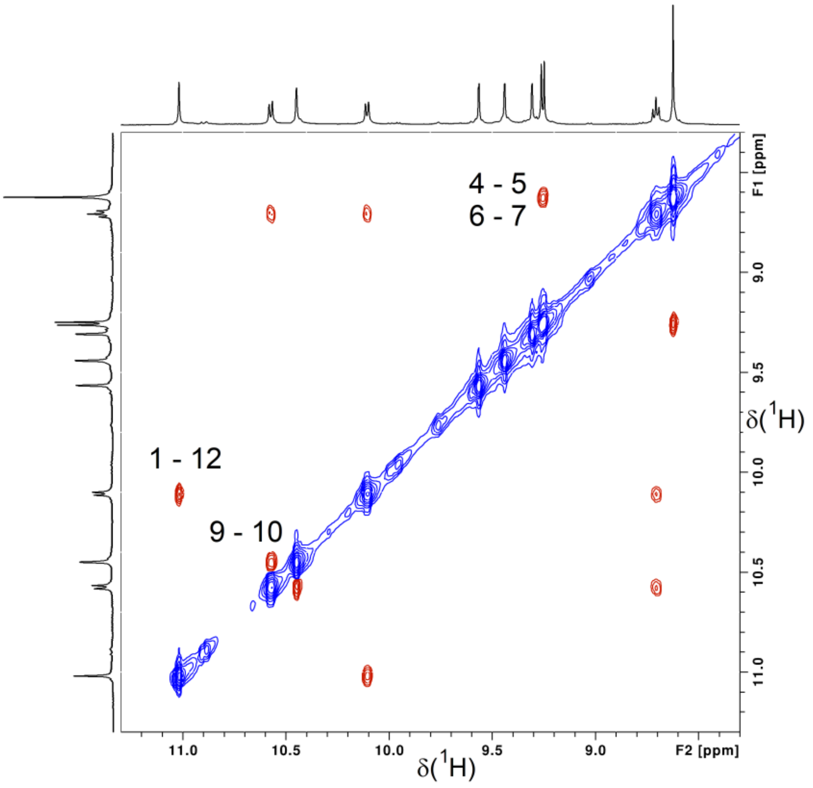

Figure S12. ROESY spectrum of **1b** (region) with assigned correlations due to spatial neighbourhoods in cove (1 - 12 and 9 - 10) and *peri* regions (4 - 5 and 6 - 7) (toluene-d_8).

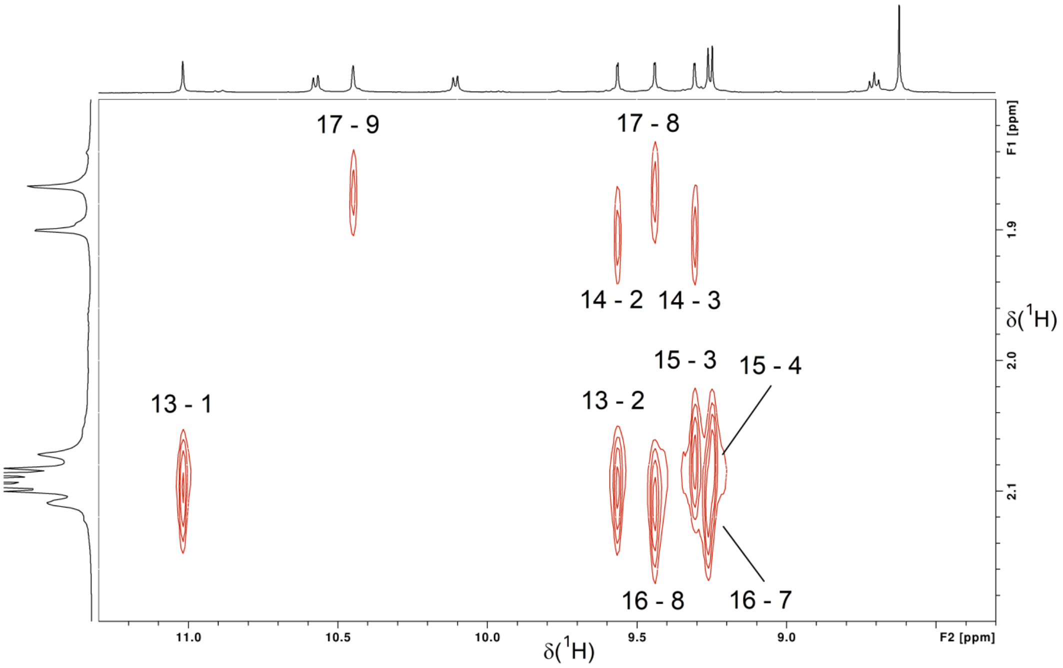

Figure S13. ROESY spectrum of **1b** (region) showing the correlations of *tert*-butyl signals to neighbouring aromatic protons (toluene-d_8).

2.5 UV–vis absorption and emission spectra of 5a, 5b, 1a, 1a′ and 1b

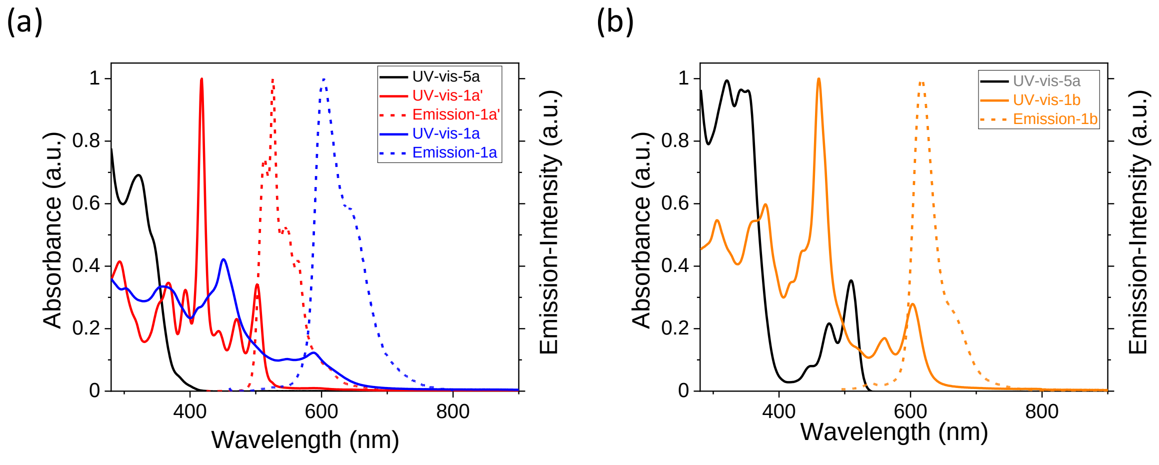

Figure S14. UV–vis absorption and emission spectra of (a) **5a**, **1a** and **1a′** in anhydrous DCM (10^{-5} M) and (b) **5b, 1b** in THF solution (10^{-5} M).

The precursor **5a** shows an absorption maximum at 321 nm with a small shoulder at 345 nm. Compared with precursor **5a**, compounds **1a′** and **1a** display distinct red-shift due to their extended aromatic cores, exhibiting the absorption maxima at 501 nm and 586 nm, respectively. The optical bandgaps of **1a′** and **1a** are calculated to be 2.13 and 1.85 eV, respectively, based on the onset of the UV–vis absorption. The emission spectrum of **1a′** exhibits peaks at 527 and 549 nm with a Stokes shift of 21 nm, and the emission spectrum of model compound **1a** exhibits peaks at 600 and 649 nm with a Stokes shift of 20 nm. The UV–vis absorption and emission peaks of **1a** show the larger red-shift than that of **1a′**, respectively, which also elucidate the smaller conjugation length of partial cyclized **1a′**.

2.6 Optimized structure of 1a′

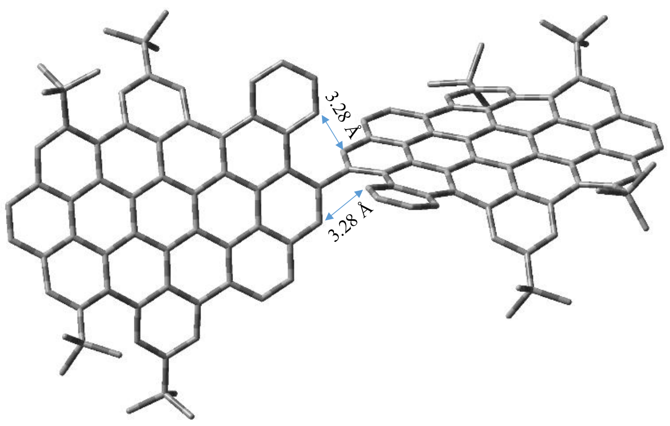

Figure S15. Optimized structure of **1a′** by DFT calculation at the HSE06/6-31G(d) level.

3. Detailed synthetic procedure and characterization data of P1 and the cMGNR

3.1 Synthesis and characterization of P1

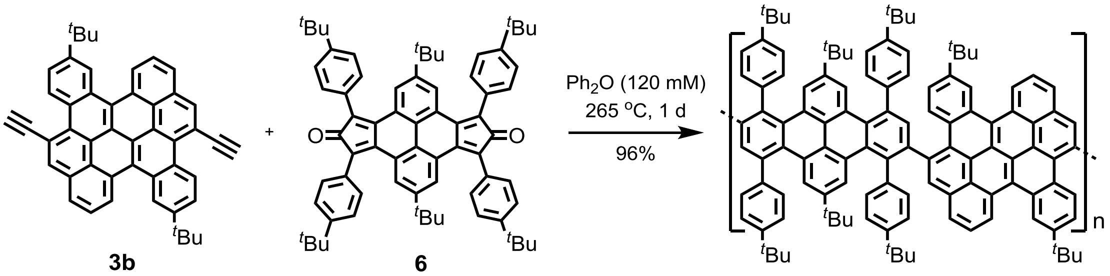

3b 6 P1

The polymerization was carried out via A_2B_2-type Diels-Alder polymerization. A degassed solution of monomer **3b** (20.0 mg, 32.7 µmol) and monomer **6** (31.0 mg, 32.7 µmol) in diphenyl ether (0.27 mL, 120 mM) was refluxed for 24 h using a heating mantle. The purple color disappeared and the solution turned pale yellow, indicating the completion of the polymerization. After cooling down to room temperature, methanol was added. The precipitates were collected by filtration with a membrane filter to obtain a crude polymer (**P1**) as a pale-yellow solid (47.2 mg, 96%). The crude **P1** was fractionalized to three fractions by using recycling GPC ($CHCl_3$ as eluent), which were characterized with analytical GPC (Figure S16) and MALDI-TOF MS (Figure S17).

(a)

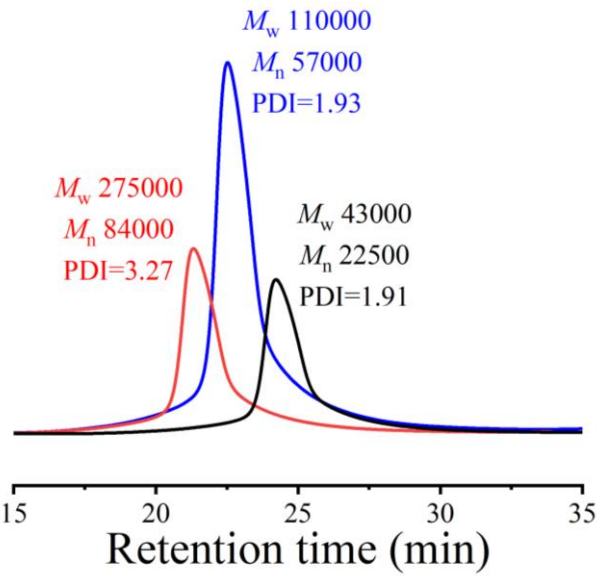

(b)

Fraction	wt (%)	M_n (g/mol)	M_w (g/mol)	PDI	Average degree of polymerization	Average length of resultant GNR
**1**	**18**	**84000**	**275000**	**3.27**	**56**	**105 nm**
**2**	**75**	**57000**	**110000**	**1.93**	**38**	**71 nm**
**3**	**7**	**22400**	**43000**	**1.91**	**15**	**28 nm**

Figure S16. (a) GPC curves of fractions 1, 2 and 3. (b) GPC results of fractions 1, 2, 3. The average degree of polymerization is calculated from molecular weight of the repeat unit (1502 g/mol) and M_n. The average length of the corresponding **cMGNR** from the different fraction of **P1** is estimated from the degree of polymerization and the length of the repeat unit (1.87 nm based on the DFT simulation). Polystyrene was used for calibration.

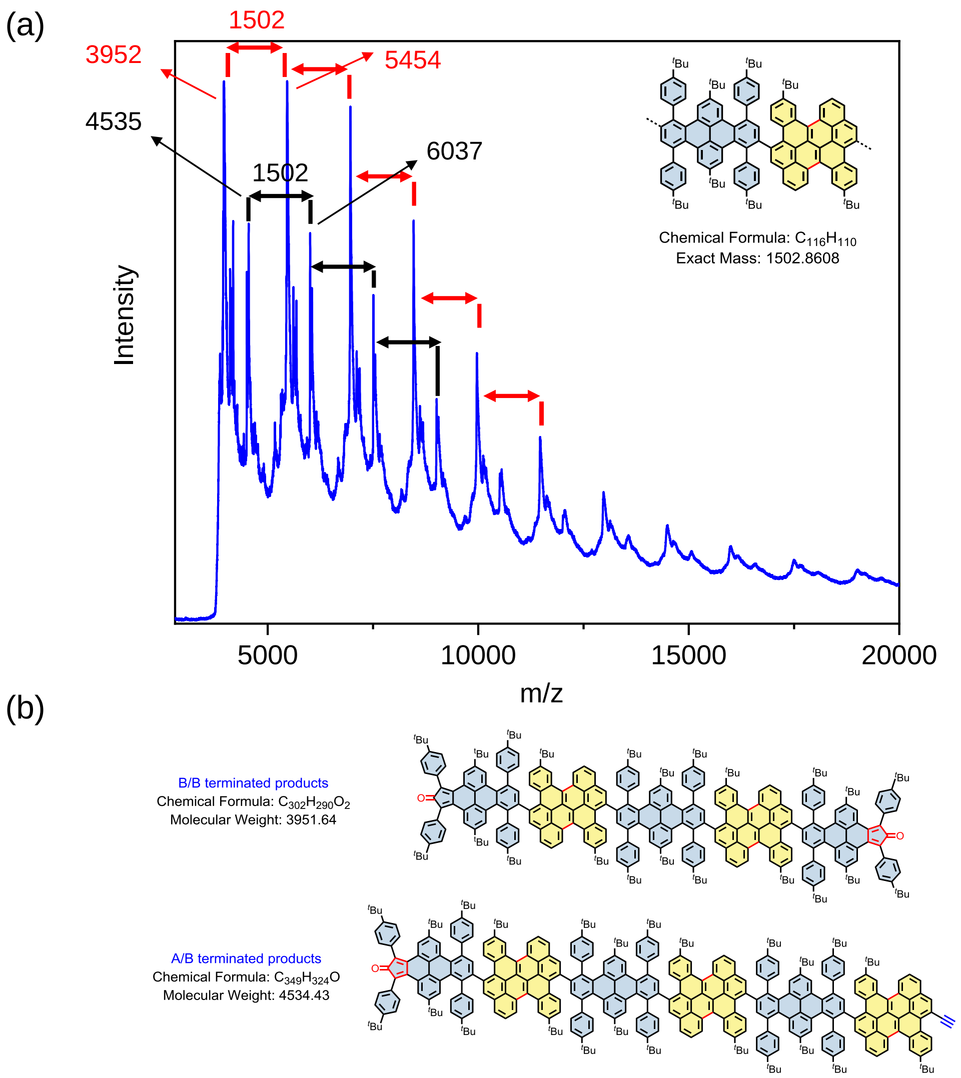

Figure S17. (a) MALDI-TOF MS characterization of **P1** (fraction 2); (b) the chemical structures of one B/B-terminated product (up) and one A/B-terminated product (bottom).

A sequence of peaks with an interval of m/z ≈ 1502 was observed peaks in Figure S17a. In principle, it should show three main signals from the A/A, A/B and B/B terminated products. However, we could only assign the A/B (label with black color) and B/B (label with red color) terminated products (see the structures that assigned to the first two peaks in Figure S17b). Therefore, we supposed there might be two possibilities that we did not observe the A/A terminated product. The first one is the difficulty in perfectly controlling the stoichiometry of the two different monomers used for the A_2B_2-type polymerization. In our case, if the amount of bisdienophile monomer (AA) is slightly less than the bisdiene monomer (BB), it will be difficult to identify the A/A terminated product due to the relatively low ratio in comparison with A/B and B/B. The second possibility is the fragmentation of the A/A-terminated product under the MALDI-TOF measurement.

3.2 Synthesis and characterization of the cMGNR

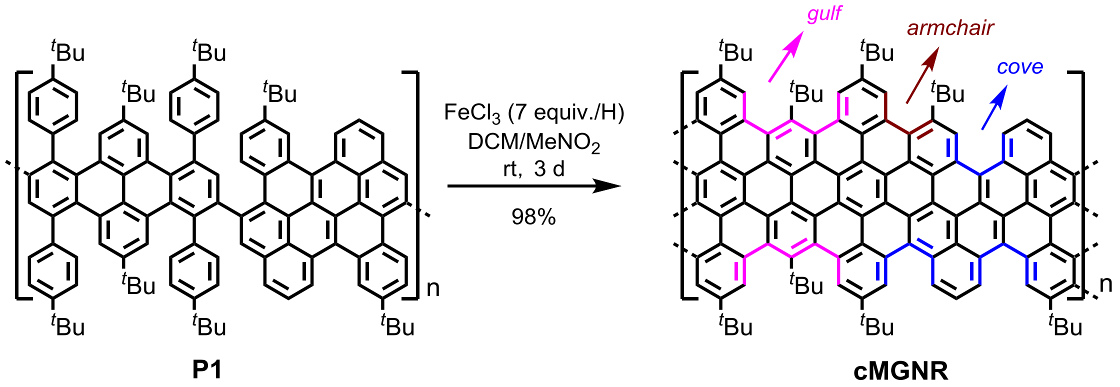

20.0 mg of **P1** (fraction 2) was dissolved in 60 mL degassed DCM. Then the solution of iron(III) chloride (257 mg, 7.0 equiv. for one hydrogen to be removed) in 2.4 mL nitromethane was added dropwise. Subsequently, a stream of Ar saturated with dichloromethane was passed through the mixture for 24 h. The mixture was stirred at room temperature for another 2 days. After the reaction, an excess amount of methanol was added to produce precipitate. The precipitate was collected by extraction filtration, washed with water and methanol for 5 cycles. After vacuum drying, 19.2 mg dark powder (**cMGNR**) was obtained with the yield of 98%.

4. Solid-state NMR characterization of P1 and the cMGNR

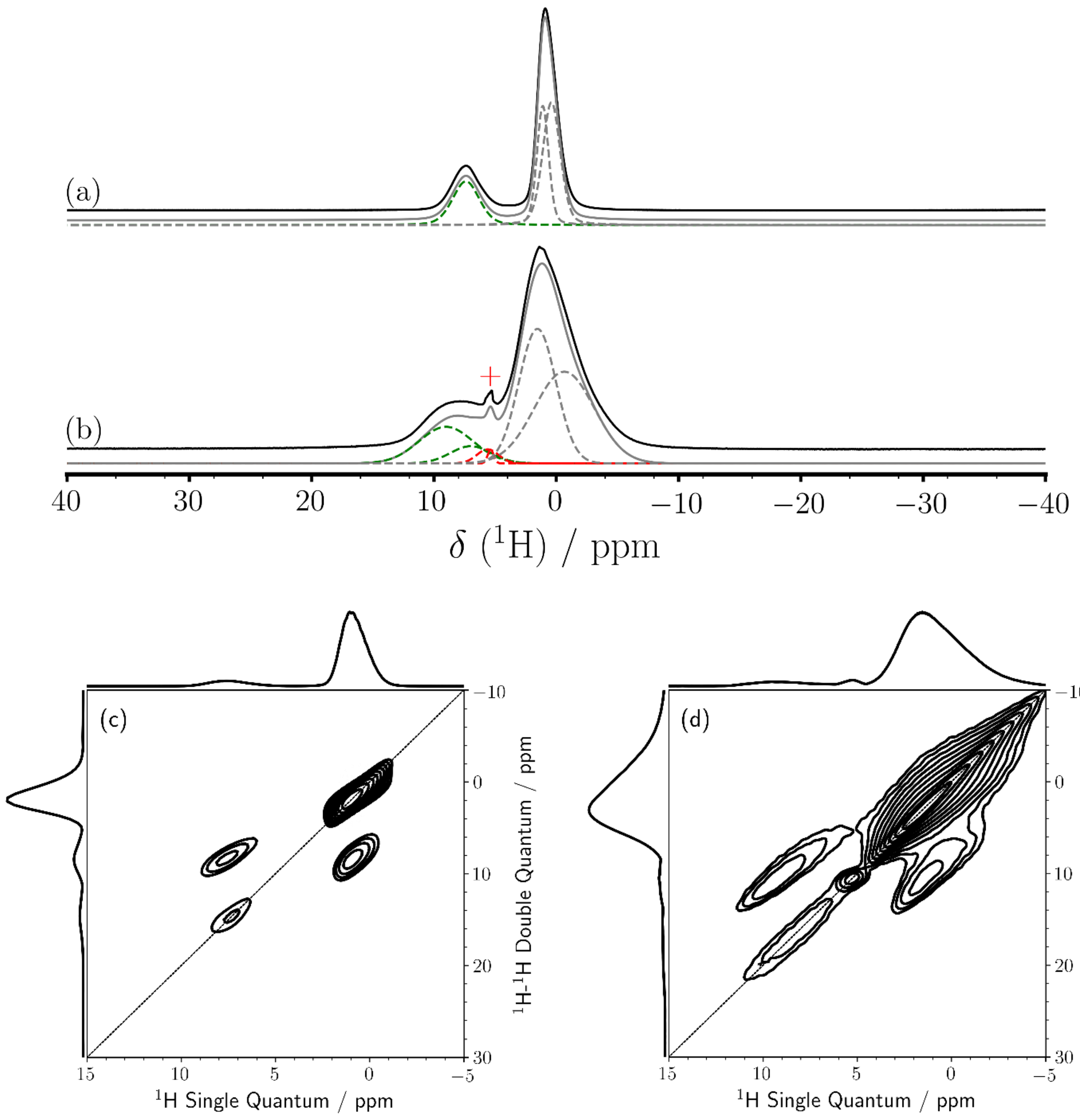

Figure S18. (a,b) ^{1}H MAS NMR and (c,d) 2D ^{1}H-^{1}H DQ-SQ NMR correlation spectra of (a,c) **P1** and (b,d) the **cMGNR** recorded at 11.76 T employing a spinning frequency of (a,c) 50.0 and (b,d) 62.5 kHz under ambient conditions. A rotor synchronized spin echo (τ_{echo}= $2\tau_r$=40 or 32 µs) was applied to eliminate the background signals. The cross marks a small impurity. The corresponding fit is shown as a gray solid line with the contributions of the aromatic (green), aliphatic (gray) protons, and the impurity (3 %, red) all highlighted in colored dashed lines. Eight rotor periods (160 µs and 128 µs) of back-to-back (BaBa) DQ recoupling and reconversion blocks were applied. The direct and indirect ^{1}H dimensions show the skyline projection. The diagonal lines are guides to the eye indicating the location of ^{1}H-^{1}H auto-correlation signals.

The solid-state ^{1}H MAS NMR spectra recorded at 11.76 T at 50.0 and 62.5 kHz MAS for **P1** and the **cMGNR** are depicted in Figure S18a and S18b, respectively. A spin echo (τ_{echo}= $2\tau_{r}$=40 or 32 µs) was applied to eliminate the probe background signal. For **P1**, two well separated ^{1}H signals can be observed whereas for the **cMGNR** the ^{1}H signals are overlapping due to the increased linewidth as expected for the sample after dehydrogenation. A small impurity at 5.2 ppm can be observed which may be caused by residual solvent (e.g., DCM). A deconvolution of the ^{1}H signals yields a ratio of 25/75% for the aromatic vs. aliphatic proton signals for **P1**. Theoretically, a ratio of 33:67% for 26 aromatic and 72 aliphatic protons can be expected. The slightly higher fraction of aliphatic protons in **P1** cannot be explained by remaining educts as the fraction of aliphatic protons in **3b** (56%) and **6** (73%) is lower than the observed fraction, which also excludes end-group effects. However, the additional amount of aliphatic protons does not persist through the dehydrogenation, resulting in formation of the **cMGNR**, as the observed ratio of 20% aromatic protons and 80% aliphatic proton is in good agreement with the theoretical ratio (18:82%). The experimental and theoretical ratios for **P1** and the **cMGNR** are summarized in Table S1. The 2D ^{1}H-^{1}H DQ-SQ NMR correlation spectra in Figure S18c and S18d for **P1** and the **cMGNR** reveal ^{1}H-^{1}H cross-correlation signals between the aromatic and aliphatic protons, respectively. For the **cMGNR**, no ^{1}H-^{1}H cross-correlation signal of the impurity at 5.2 ppm is present. In addition, analogously to recent investigations of GNR samples, the width of the ^{1}H-^{1}H auto-correlation signal of the aromatic protons can be taken as an indication for the aggregation of GNR. The low span of only up to ~11/22 ppm of the aromatic ^{1}H-^{1}H auto-correlation signal indicates reduced π-π interactions compared to planar GNR samples.[2, 8]

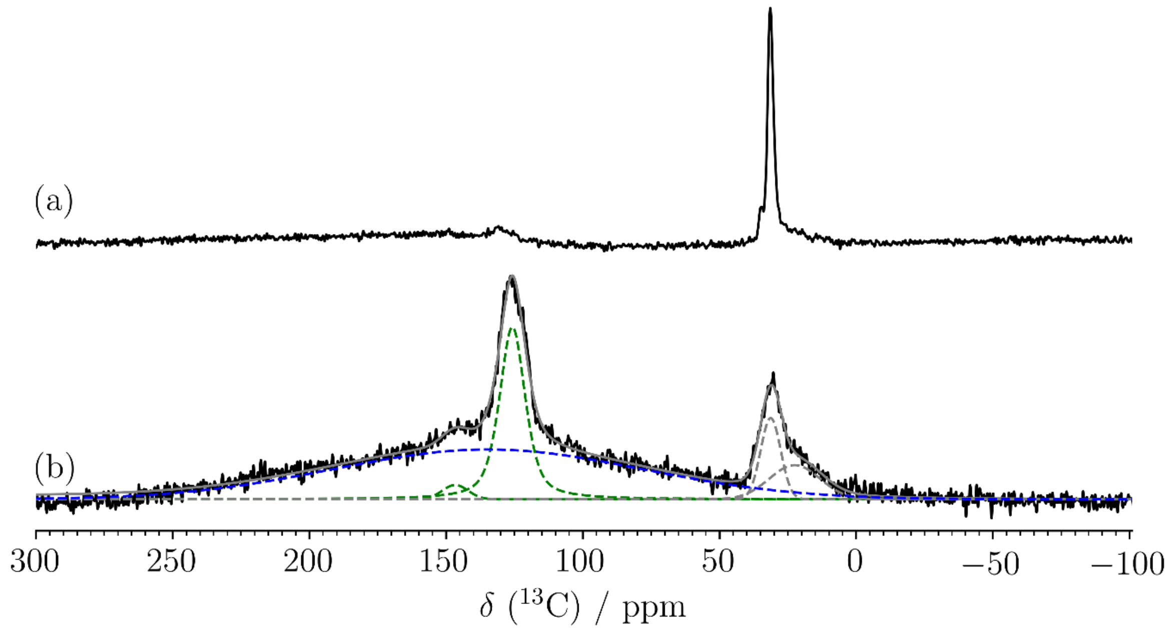

Figure S19. $^{13}C\{^1H\}$ MAS spectra of (a) **P1** and (b) the **cMGNR** recorded at 11.76 T employing (a) 50.0 and (b) 62.50 kHz MAS. The corresponding fit is shown as gray line with the contributions of the single components as dashed green (aromatic ^{13}C), gray (*tert*-butyl ^{13}C), and blue (background signal) lines.

Table S1. Comparison of the fitting results with the theoretical values for ^{13}C and ^1H for **P1** and the **cMGNR**.

		theoretical		*experimental*	
Integral region		aromatic	aliphatic	aromatic	aliphatic
^1H	P1	33 %	67 %	25 %	75 %
	cMGNR	18 %	82 %	20 %	80 %
^{13}C	P1	72 %	28 %	-[a]	-[a]
	cMGNR	72 %	28 %	68 %[b]	32 %[b]

[a] The $^{13}C\{^1H\}$ MAS NMR spectra are not quantitative for the model compound.

[b] The background signal was included into the fit but not into the ratio of aromatic vs. aliphatic carbon atoms.

The $^{13}C\{^1H\}$ MAS NMR spectra also recorded at 11.76 T of **P1** and the **cMGNR** are depicted in Figure S19. Whereas the applied recycle delay for the precursor is expected to be insufficient for a quantitative analysis, previous studies of similar GNR compounds were possible with shorter recycle delays. In this study, even a recycle delay of 22 s was not sufficient for quantitative analysis as the ^{13}C signals of the aromatic carbon atoms still

increase in intensity. Thus to obtain a reasonable signal-to-noise ratio for the $^{13}C\{^1H\}$ MAS NMR spectrum of the **cMGNR** longer recycle delays were excluded. Nevertheless, the intensity ratio of aromatic and aliphatic carbon atoms with 68 and 32 %, respectively, is close to the theoretical expected values for the **cMGNR**, see Table S1. Moreover, as the intensity of the aliphatic carbon atoms is constant for the applied recycle delay, the experimental intensity ratio should approach the theoretical value. Note that the ^{13}C signals which are broadened by paramagnetic relaxation effects cannot be distinguished from the background signal (also included into the fit).

5. DFT calculations

All computations have been performed with the Gaussian16 software,[9] at the DFT level of theory using the range-separated screened HSE hybrid functional[10] with the Pople's 6-31G(d) basis set[11]. Geometry optimizations have been performed on all the short oligomers, followed by TD-DFT single point calculations to obtain the absorption spectra. In order to create the repeating unit for the the **cMGNR**, the model systems (or similar monomers) were cut in different ways. The geometries, electronic and optical properties of model compounds and short oligomers have been all calculated. The reduced mass analysis has been performed on the optimized structure of the **cMGNR**.

5.1 Geometry of the optimized structures

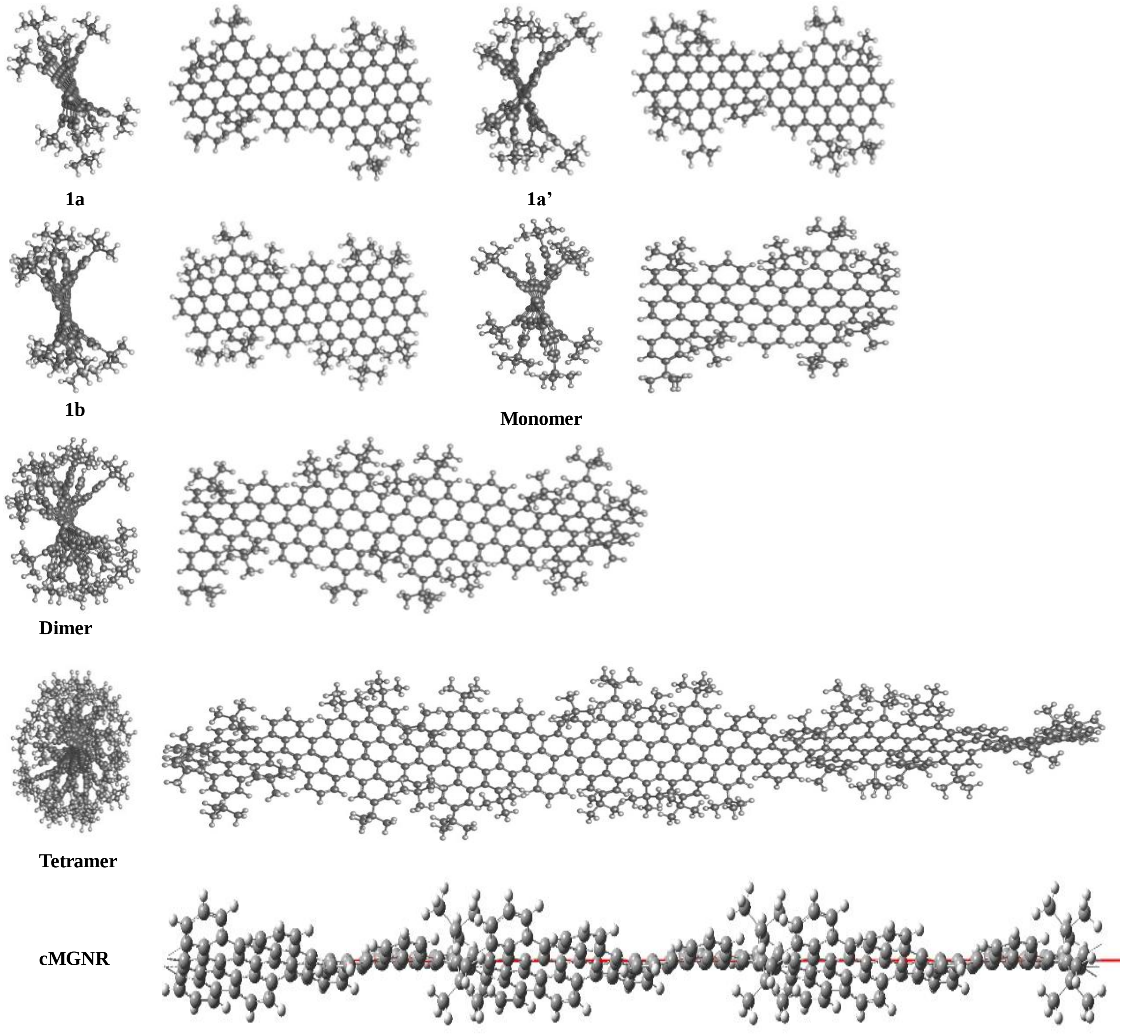

Figure S20. Optimized geometries of **1a**, **1a′**, **1b**, monomer, dimer, tetramer and the **cMGNR**.

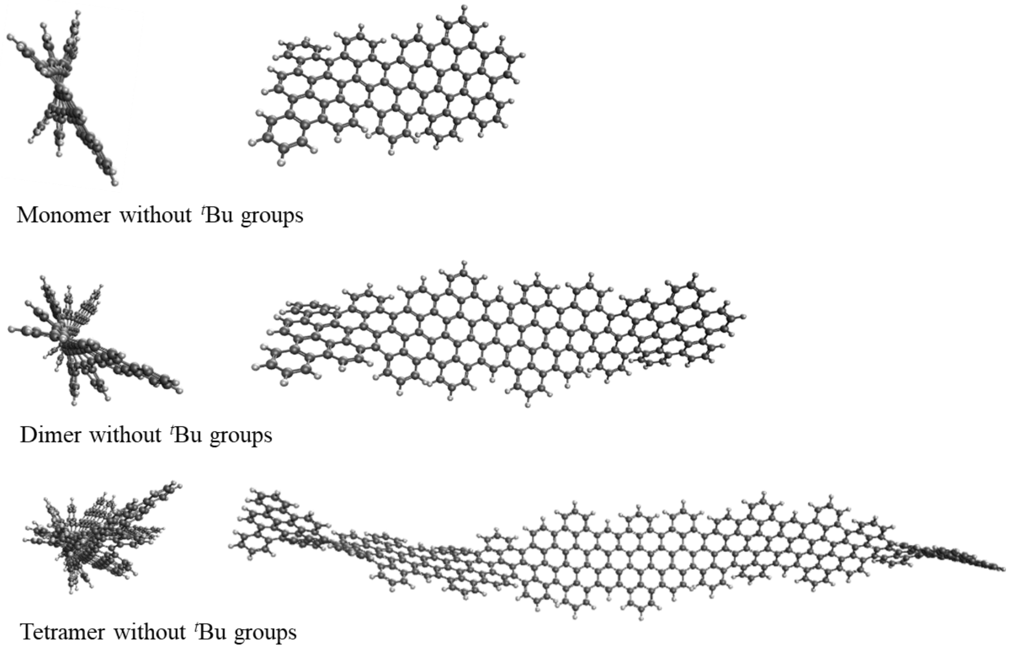

Figure S21. Optimized geometries of monomer, dimer, and tetramer without ^{t}Bu groups.

Note that it was reported that the presence of ^{t}Bu groups at the bay position of nanographenes can strongly affect the curvature of the structure, and also red shift the absorption.[12] To assess if this statement is valid for the the **cMGNRs**, we also performed the whole analysis on the same oligomers but without the ^{t}Bu groups (Figure S21). As can be seen already from the dimer, the presence of ^{t}Bu side groups strongly affects the conformation of the structure in Figure S20. This is even more pronounced when the tetramer is considered, in which a full turn of 360 degrees in the structure is obtained.

5.2 Electronic structure

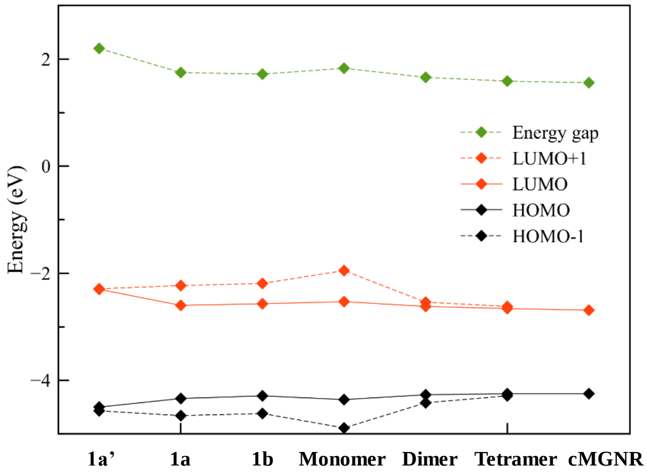

Figure S22. Calculated energy levels of **1a′**, **1a**, **1b**, monomer, dimer, tetramer and the **cMGNR**.

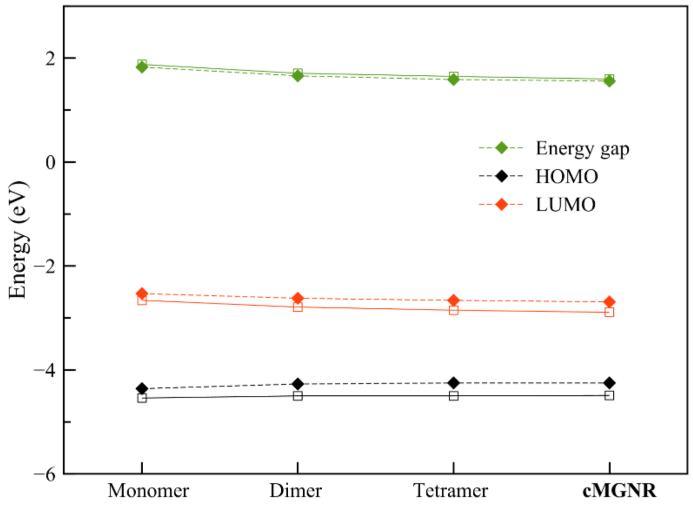

Figure S23. Comparison of the energy levels of monomer, dimer, tetramer and the **cMGNR** with ^{t}Bu groups (filled symbols) or without ^{t}Bu groups (open symbols).

The plot in Figure S22 shows the evolution of the frontier molecular orbitals with the increase of oligomer length for the structures. It seems that for this GNR the length of the ribbon has very little influence on the electronic properties, as both dimer, tetramer as well as the **cMGNR** have very similar energy and band gap, of 1.5-1.9 eV. Apart for the monomer, from the dimer onward the HOMO and LUMO energy is almost constant with respect to the length, leading to a similar value of energy gap of 1.66 and 1.59 eV for the dimer and tetramer, respectively, which converges to 1.56 eV for the **cMGNR**. The presence of a single bond in **1a′** and the associated twisting of the structure, which breaks the conjugation, has a strong impact on the energetics of the frontier orbitals. In addition, the computed optical energy gap for model compounds **1a** and **1b** have values of 1.90 and 1.87 eV, in excellent agreement with the experimental results of 1.85 and 1.81 eV, respectively.

The plot in Figure S23 depicts the differences in electronic structure between the oligomers with ^{t}Bu side groups (filled symbols) and without (open symbols). As expected, there is a mild effect on the energy levels associated with the side groups, namely a destabilization induced by the electron donating effects of the ^{t}Bu moieties. However, the fundamental gap remains unaffected, as these destabilizing effects are very similar for the occupied and unoccupied levels. Table S2 shows the shapes of their frontier orbitals.

Table S2. The shapes of the frontier orbitals of **1a′**, **1a**, **1b** as well as monomer, dimer and tetramer **(**with or without ^{t}Bu groups).

**Compounds**	HOMO	LUMO
**1a′**	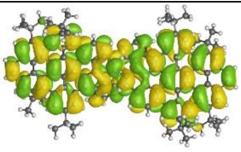	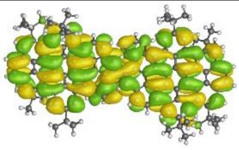
**1a**	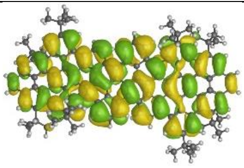	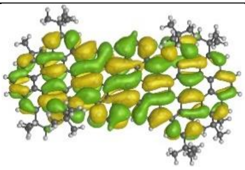
**1b**	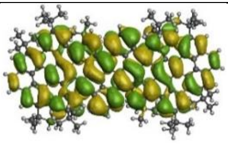	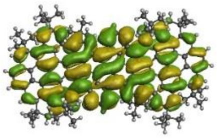
**Monomer**	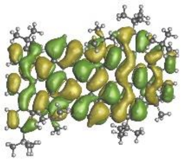	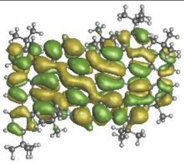
**Dimer**	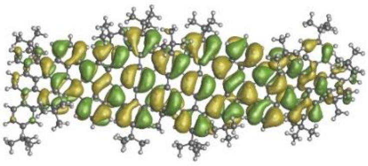	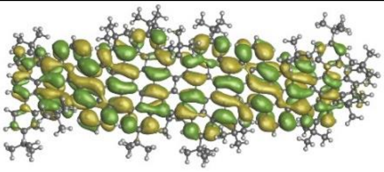
**Tetramer**	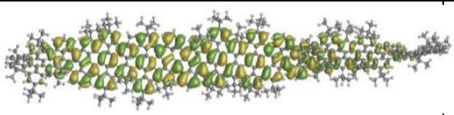	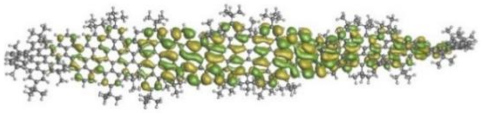
**Monomer without ^{t}Bu groups**	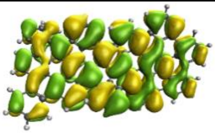	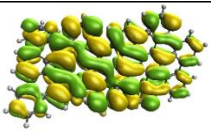
**Dimer without ^{t}Bu groups**	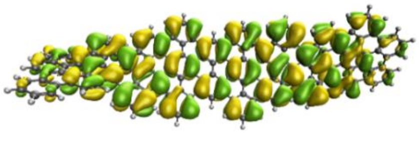	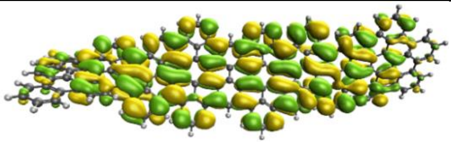
**Tetramer without ^{t}Bu groups**	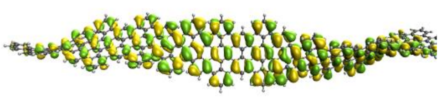	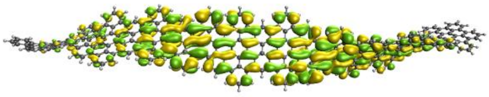

5.3 Optical absorption

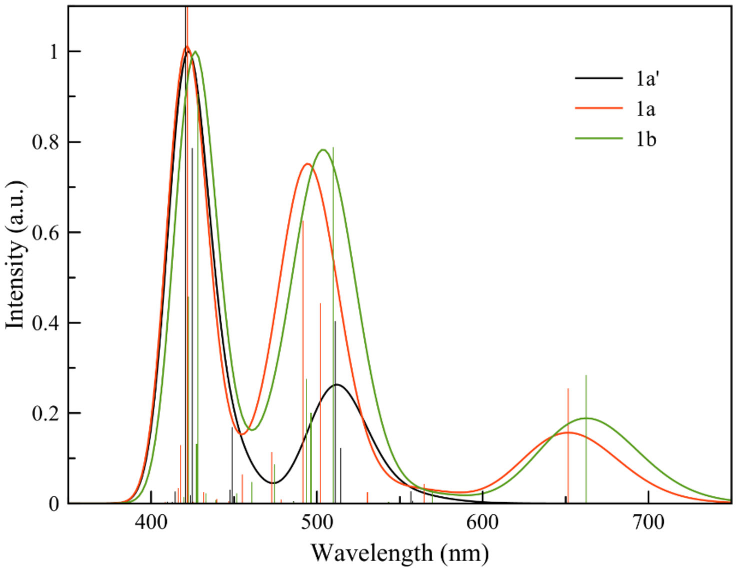

Figure S24. Calculated UV–vis absorption of **1a′**, **1a** and **1b**.

The absorption spectra of **1a′**, **1a** and **1b** have been calculated and are reported in Figure S24. As expected, a strong red shift is observed going from the **1a′** to **1b**, with the lowest absorption peak shifting from 514 to 663 nm, respectively. The addition of ^{t}Bu groups additionally redshifts the absorption by 11 nm (from **1a** to **1b**), though it does not have a significant effect on the overall shape of the spectra. The main transitions are reported in Table S3.

Table S3. The transitions at each absorption band of **1a′**, **1a** and **1b**.

	Energy (nm)	Oscillator strength (f)	Transition
**1a′**			
S1	558	0.01	H → L+1
S3	514	0.12	H-1 → L
S4	511	0.40	H-1 → L+1
S11	425	0.79	H → L+5
S13	421	1.18	H-1 → L+3
**1a**			
S1	652	0.25	H → L
S6	502	0.44	H-1 → L+1
S8	492	0.63	H-1 → L+1
S10	472	0.11	H-4 → L
S17	422	1.46	H-5 → L
**1b**			
S1	663	0.28	H → L
S6	510	0.79	H → L+2
S8	494	0.28	H-1 → L+1
S16	428	0.91	H-5 → L

S18	423	0.46	H-3 → L+1

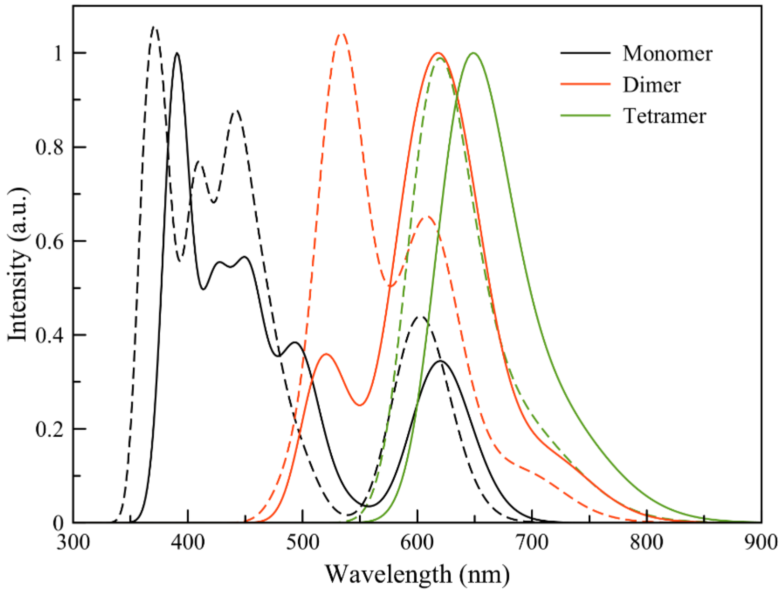

Figure S25. Calculated UV–vis absorption of monomer, dimer and tetramer with ^{t}Bu groups (solid lines) or without ^{t}Bu groups (dotted lines).

In Figure S25, the calculated absorption spectra of all the oligomers are reported. The presence of the ^{t}Bu groups indeed redshifts the absorption for all oligomers. The main transitions are reported in Table S4. In the longest investigated oligomer (tetramer), the simulated absorption spectrum shows a main absorption peak at ~640nm with a shoulder around 770nm. The presence of a dominant absorption band with a long wavelength shoulder is in line with the experimental spectrum in Fig. 3(c), though the experimental spectrum extends further in the IR. This could be due to either the presence of ribbons with longer physical lengths and lower excitation energies (unlikely in view of the saturation observed at Fig.S22) or (more likely) to scattering.

Table S4. The transitions at each absorption band of monomer, dimer and tetramer as well as their parent analogues without ^{t}Bu groups.

**monomer**	Energy (nm)	Oscillator strength (f)	Transition
1	620	0.36	H → L
4	507	0.14	H → L+1
7	456	0.30	H-3 → L<br>H → L+3
11	424	0.27	H-1 → L+1
17	389	0.58	H-2 → L+2
**dimer**			
1	722	0.14	H → L
2	705	0.05	H → L+1
5	631	0.76	H-1 → L+1
6	605	0.43	H-2 → L
14	531	0.17	H-3 → L+2
**tetramer**			
1	769	0.06	H → L
2	758	0.06	H-1 → L
5	730	0.22	H-1 → L+1
15	655	0.44	H → L+5
20	637	1.33	H-3 → L+3
**monomer without ^{t}Bu**			
1	602	0.33	H → L
4	496	0.10	H-1 → L /<br>H → L+1
6	467	0.27	H-2 → L /<br>H → L+2
7	444	0.24	H → L+3
8	438	0.36	H-3 → L
11	408	0.44	H-4 → L
16	378	0.46	H-2 → L+2
**dimer without ^{t}Bu**			
1	696	0.11	H → L
4	610	0.67	H-1 → L /<br>H → L+1
7	564	0.15	H-2 → L /<br>H → L+2
10	534	0.36	H-1 → L+3
11	533	0.65	H-3 → L
**tetramer without ^{t}Bu**			
1	742	0.05	H → L
3	718	0.01	H → L+1
5	704	0.18	H-1 → L+1
11	661	0.24	H-2 → L+2
15	627	0.47	H-3 → L+2 /<br>H-2 → L+3
17	614	1.06	H-3 → L+3

5.4 Band dispersion and effective masses

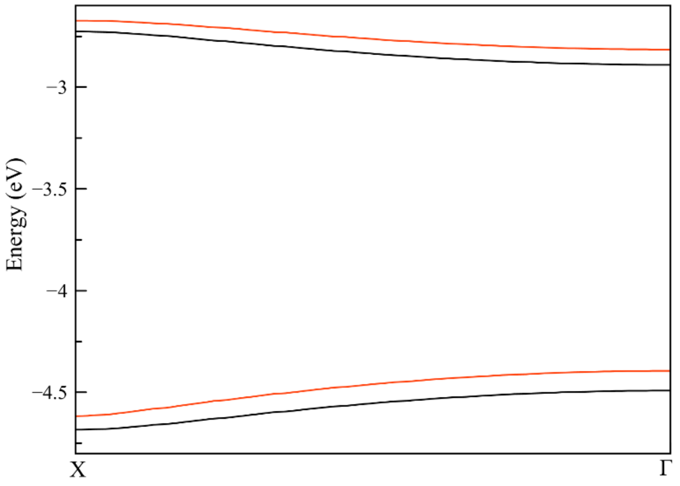

Figure S26. Calculated band structure of the **cMGNR** (orange line) and its analogue without ^{t}Bu groups (black line).

The band dispersion of the 1D **cMGNR** ribbons is reported in Figure S26. The presence of ^{t}Bu side groups seems to affect only slightly the band gap, which has value of 1.60 eV without ^{t}Bu and slightly decreases to 1.56 eV when ^{t}Bu groups are present. The main effect of the ^{t}Bu side groups is to shift the VB and CB to higher energy, as discussed above. The effective masses are relatively large, reaching 2.16 for m^*_h (effective mass of the hole) and 1.27 for m^*_e (effective mass of the electron) for the **cMGNR** with ^{t}Bu groups. A comparison between the three different curved GNRs (**cMGNR** in this work; **cGNR**[13a]; ***6*-CZGNR-(2,1)**[13b]): the ***6*-CZGNR-(2,1)** has the highest dispersion of VB and CB, resulting in a low effective mass ($m^*_h = m^*_e = 0.17\ m_0$), while for the **cMGNR** the VB and CB are rather flat (Figure S27). In turn, this flat band dispersion of **cMGNR** translates into having higher values for the effective mass.

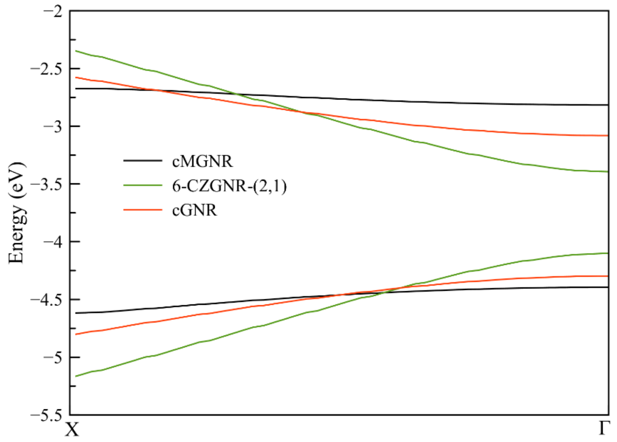

Figure S27. Comparison of the calculated band structure of three different curved GNRs.

5.5 IR spectrum

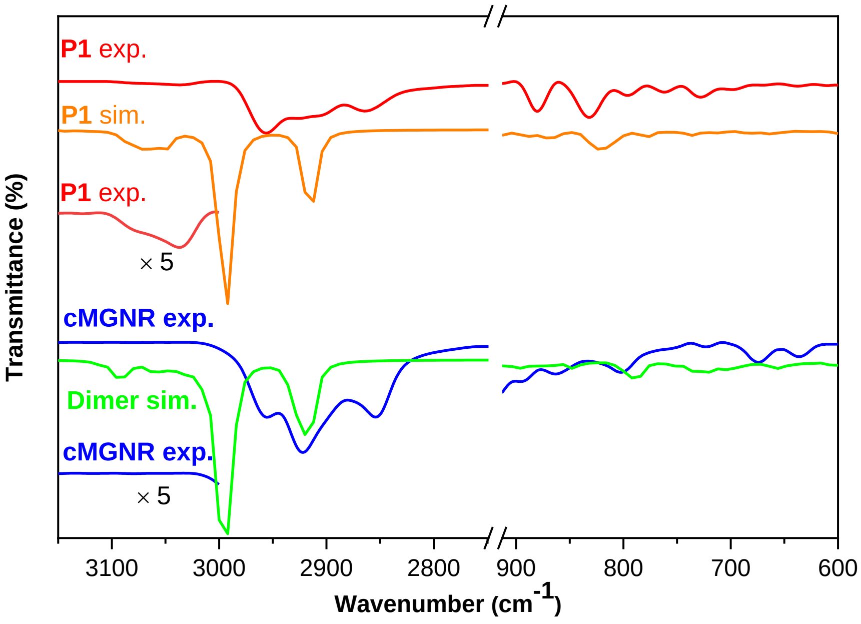

Figure S28. The calculated IR spectrum of the dimer and polymer.

The IR spectrum was computed on both the dimer and its precursor structure. Since there is little dependence of electronic properties on the length, these short oligomers were considered as model for the **cMGNR**.

There is a good agreement between the computed and the experimental IR spectra. At low wavenumber, in the 500-900 cm^{-1} range, several peaks of interest are considered. For the dimer, the peak at 656 cm^{-1} (exp 674 cm^{-1}) relates to the in plane bending modes of the carbon atoms, 720 cm^{-1} relates to the out-of-pane bending modes of the carbon atoms of the ribbon. The next peak at 780 cm^{-1} (exp 802 cm^{-1}) relates to the out-of-plane bending of the phenyl rings, at the edges of the ribbon. Next peak at 877 cm^{-1} (exp 863 and 892 cm^{-1}) relates to the breathing modes of the carbon atoms of the ribbon (which is not present for the polymer). In the high wavenumber region, the peak at 2913 (2991) cm^{-1} relates to the symmetric (asymmetric) stretching of the methyl hydrogens on the ^{t}Bu groups, and the shoulder at 3091 cm^{-1} are associated to the symmetric stretching modes of hydrogen atoms of the phenyl rings. Compared to the polymer, the signals from the typical aromatic C–H stretching vibrations (broad peaks between 3047 and 3075 cm^{-1} for polymer) were significantly decreased, indicating the efficient “graphitization.

For the polymer, the peak at 773 cm^{-1} (exp 796 cm^{-1}) relates to the out-of-plane bending of the phenyl rings without ^{t}Bu groups; 809 cm^{-1} (exp 832 cm^{-1}) relates to the in plane bending of the phenyl rings without ^{t}Bu groups and at 822 cm^{-1} to the hydrogen atoms out-of-plane bending of the phenyl rings which are not graphitized. Next peak at 878 cm^{-1} (exp 879 cm^{-1}) relates to the asymmetric bending of the hydrogen atoms of the phenyl rings which are not graphitized. In the high wavenumber region, the peak at 2911 (2991) cm^{-1} relates to the symmetric (asymmetric) stretching of the methyl hydrogens on the ^{t}Bu groups.

6. Terahertz spectroscopic study of the cMGNR

Drude-Smith (DS) model and fitting

The Drude-Smith (DS) model, modified from the free-carrier Drude model, has been employed widely to infer the charge transport properties in nanostructured semiconductors (*i.e.*, graphene nanoribbon).[13a, 14] The model reads:

$$\sigma(\omega) = \frac{\omega_p^2 \varepsilon_0 \tau}{1 - \mathrm{i}\omega\tau}\left(1 + \frac{c}{1 - \mathrm{i}\omega\tau}\right)$$

where τ, ω_p and ε_0 are the Drude-Smith charge scattering time, the plasma frequency, and vacuum permittivity, respectively. The plasma frequency ω_p is relative to the density of excited charge carriers. Here a parameter c is introduced to characterize the backscattering probability for the transport of free charge carriers, due to *e.g.*, structural confinement; c is ranging from 0 (isotropic scattering) and -1 (fully backscattering). We estimate the mobility of the charge carriers in the *dc* limit following $\mu=\frac{e\tau}{m^*}(1 + c)$, with m^* as the reduced effective mass of charge carriers, following: $\frac{1}{m^*} = \frac{1}{m_\mathrm{e}^*} + \frac{1}{m_\mathrm{h}^*}$. Here m_e^* and m_h^* stand for the effective mass for electrons and holes inferred from DFT calculations.

7. NMR spectra

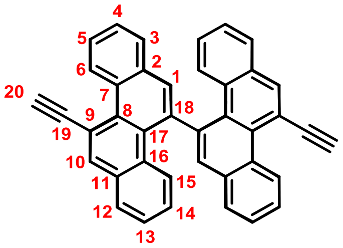

3a

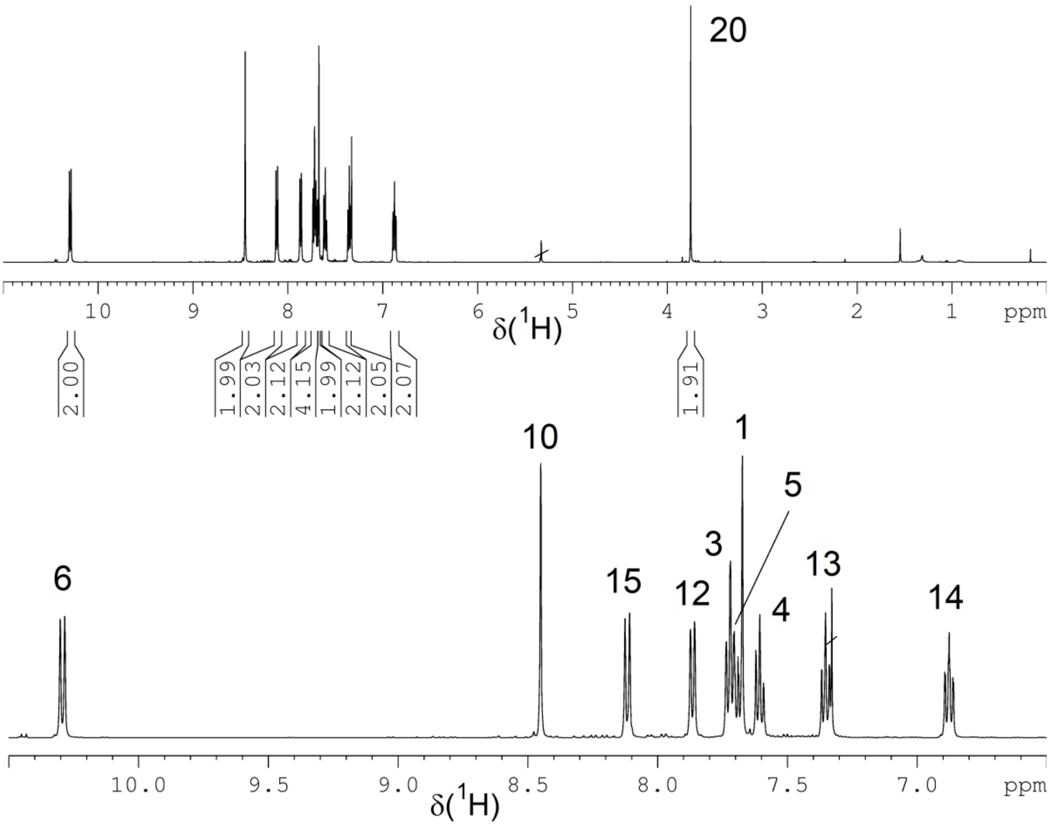

Figure S29. ^{1}H NMR spectrum of **3a** (solvent: CD_2Cl_2).

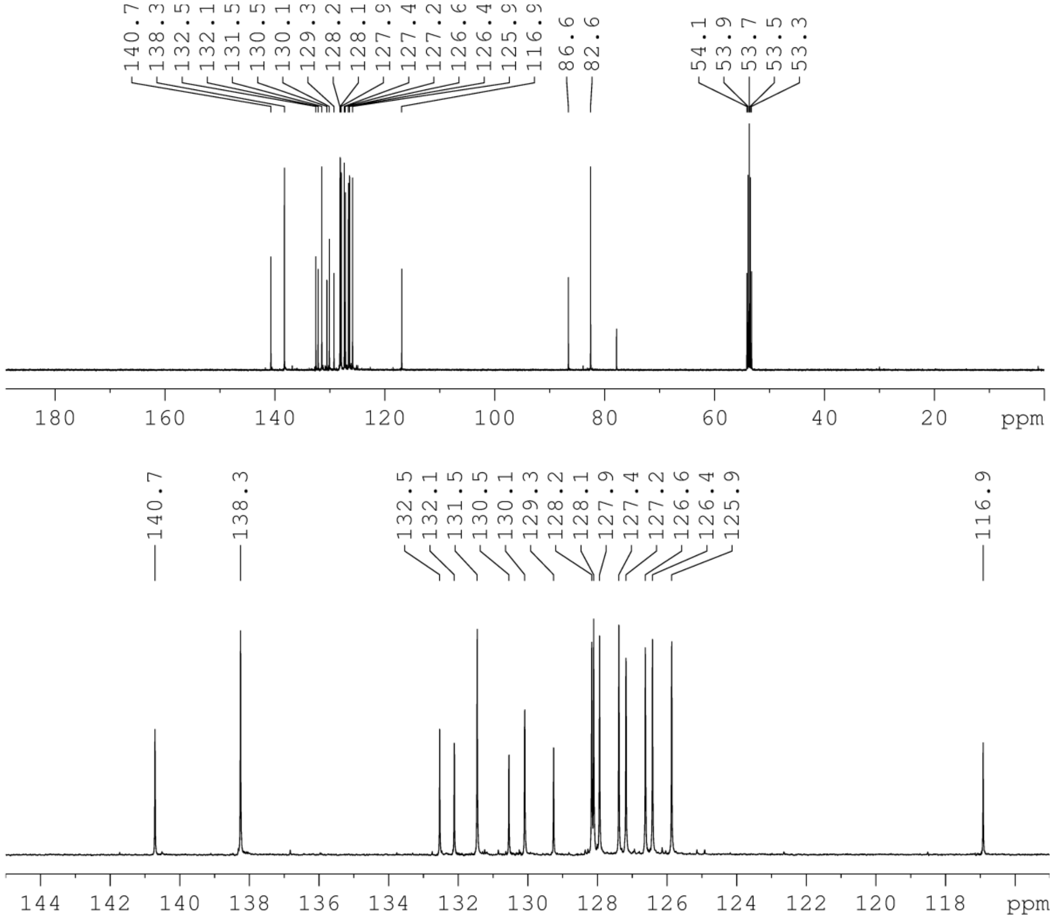

Figure S30. ^{13}C NMR spectrum of **3a** (solvent: CD_2Cl_2).

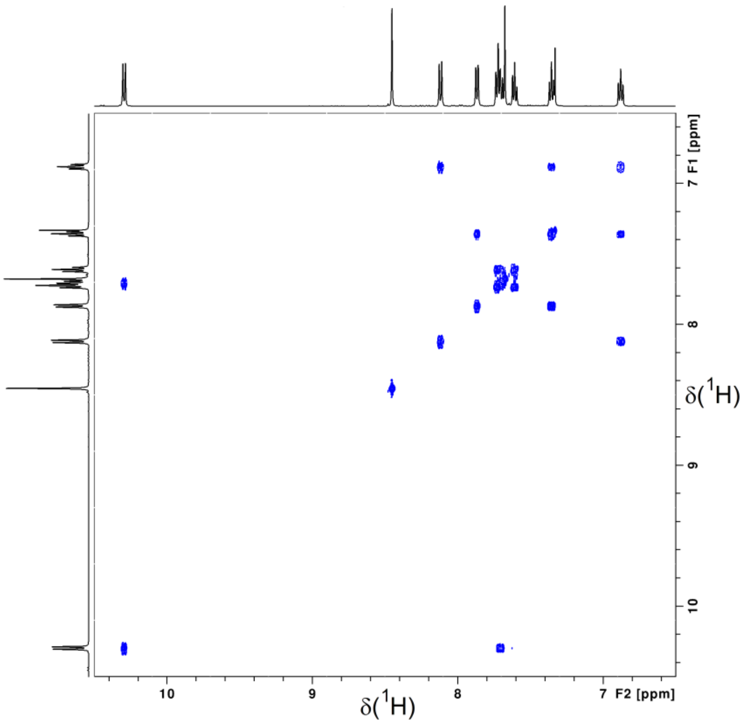

Figure S31. COSY spectrum of **3a** (solvent: CD_2Cl_2).

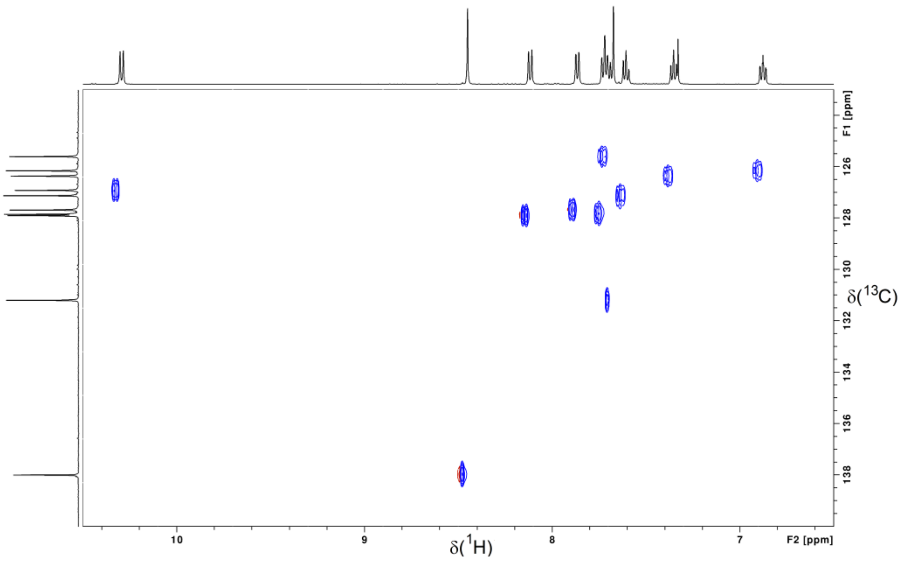

Figure S32. HSQC spectrum (region) of **3a** (solvent: CD_2Cl_2).

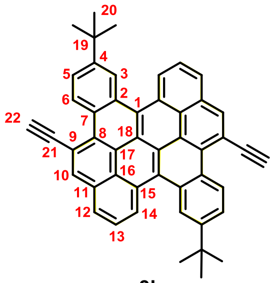

3b

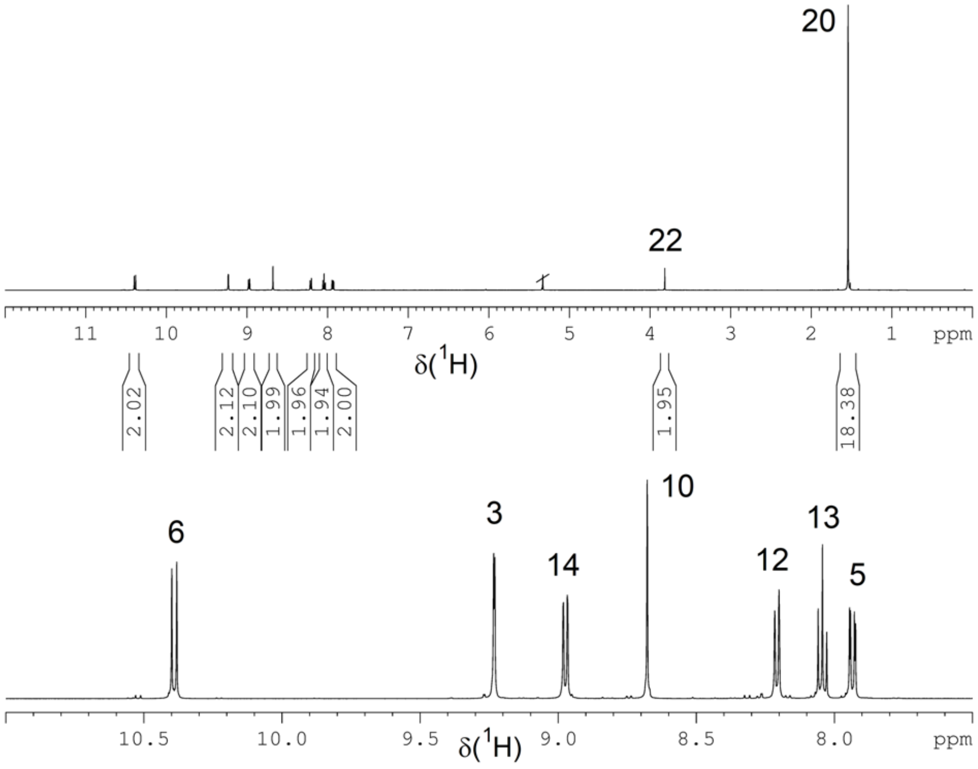

Figure S33. ^{1}H NMR spectrum of **3b** (solvent: CD_2Cl_2).

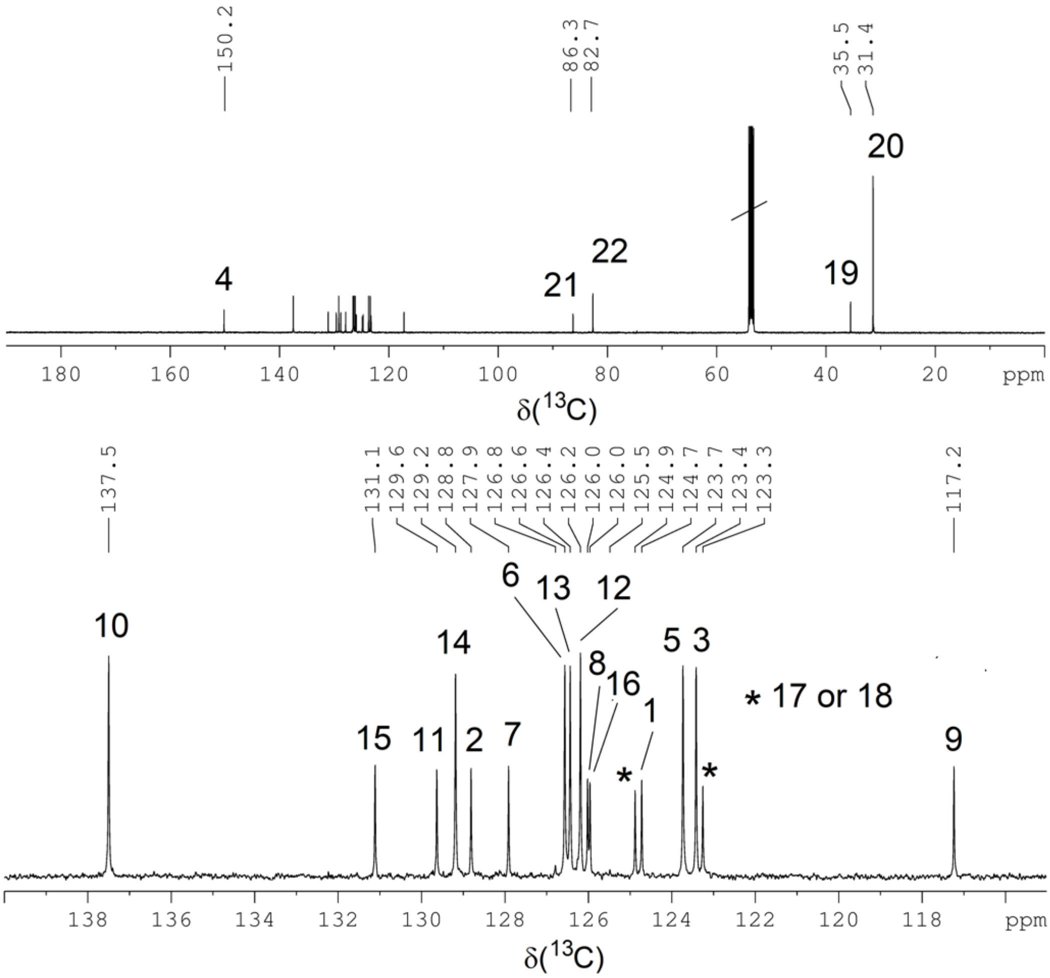

Figure S34. ^{13}C NMR spectrum of **3b** (solvent: CD_2Cl_2).

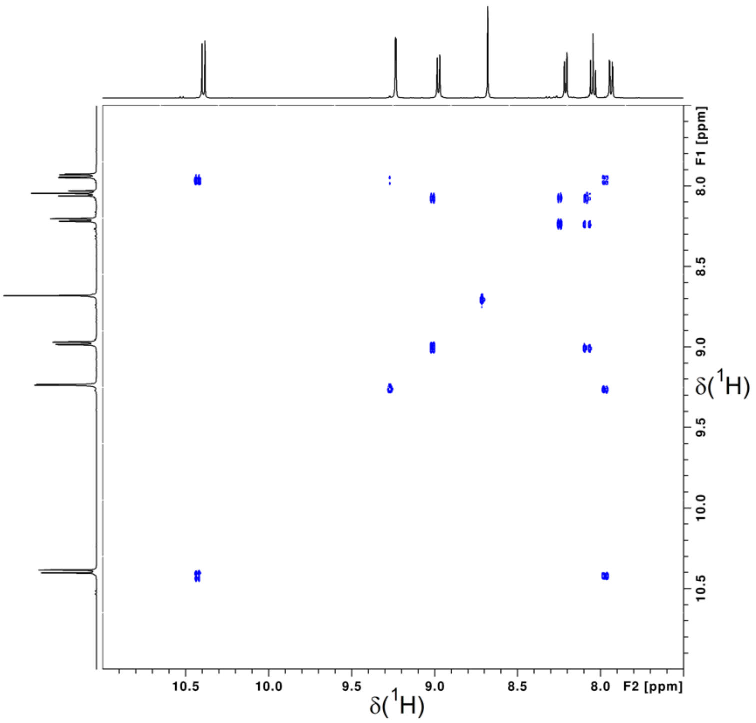

Figure S35. COSY spectrum of **3b** (solvent: CD_2Cl_2).

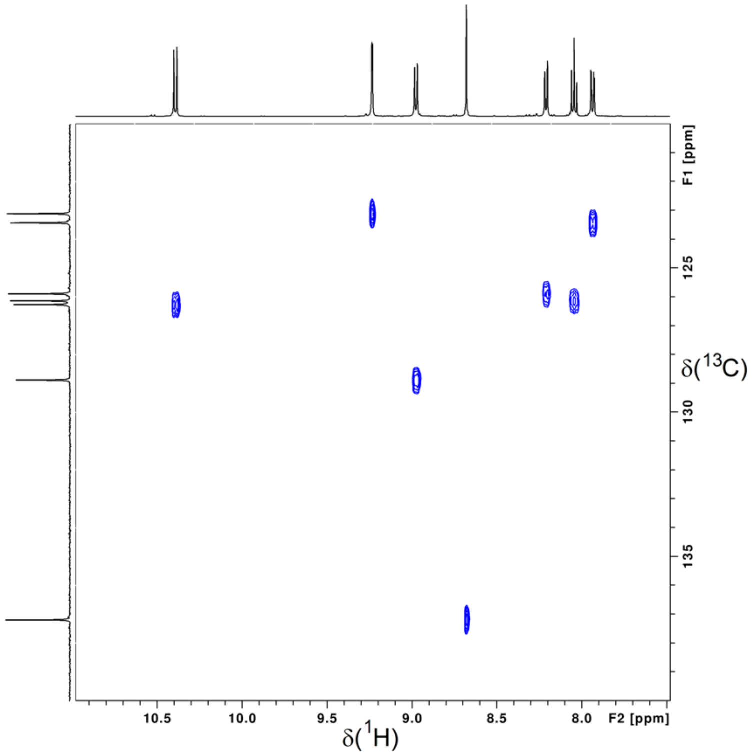

Figure S36. HSQC spectrum (region) of **3b** (solvent: CD_2Cl_2).

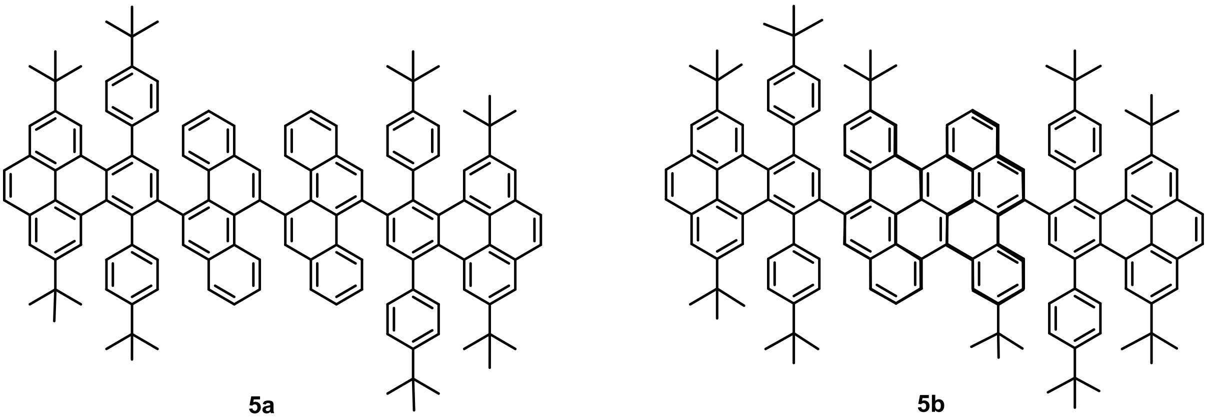

5a **5b**

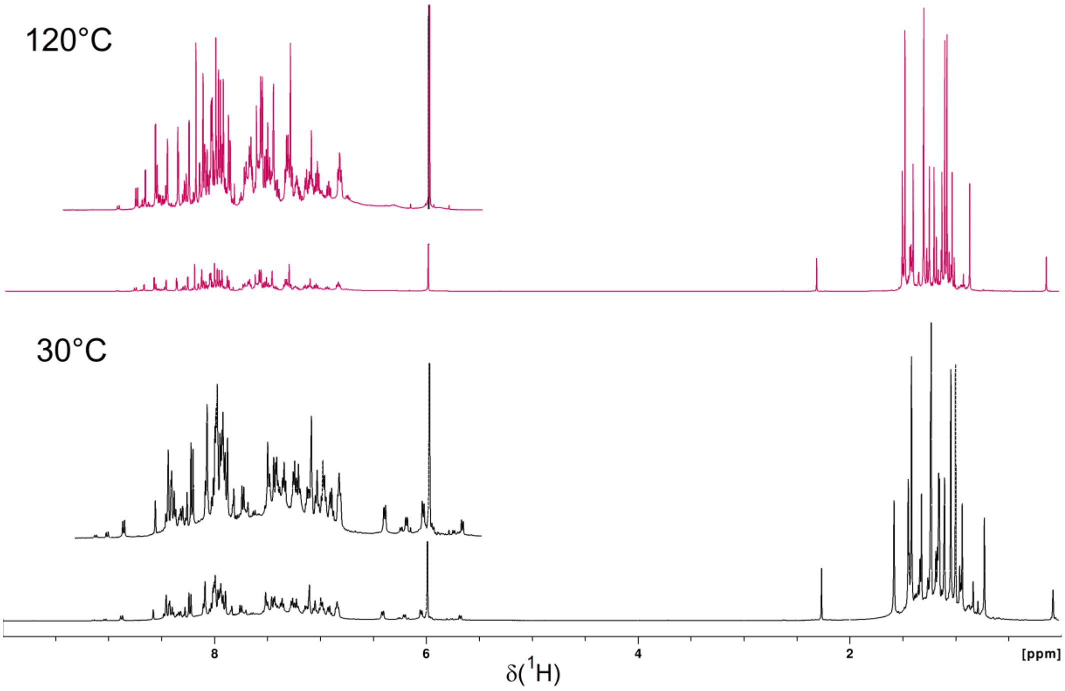

Figure S37. ^{1}H NMR spectrum of **5a** at 30°C and at 120°C (solvent: $C_2D_2Cl_4$).

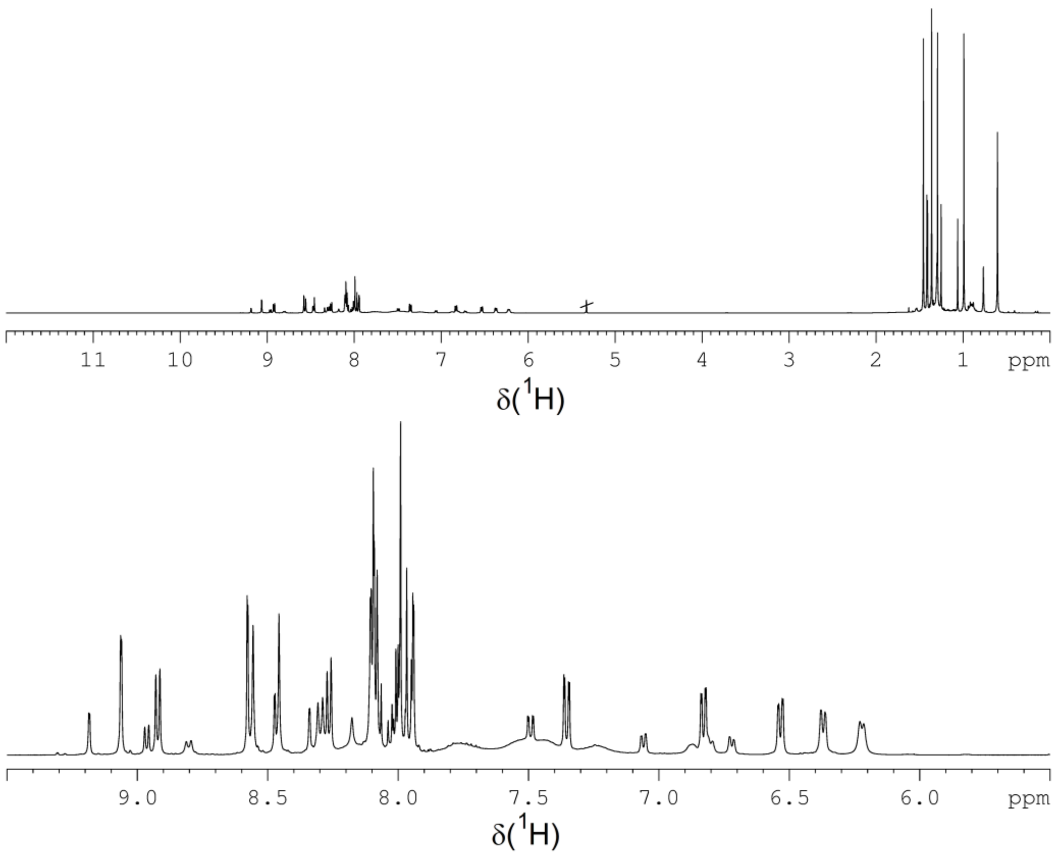

Figure S38. ^{1}H NMR spectrum of **5b** and region of aromatic protons (solvent: CD_2Cl_2).

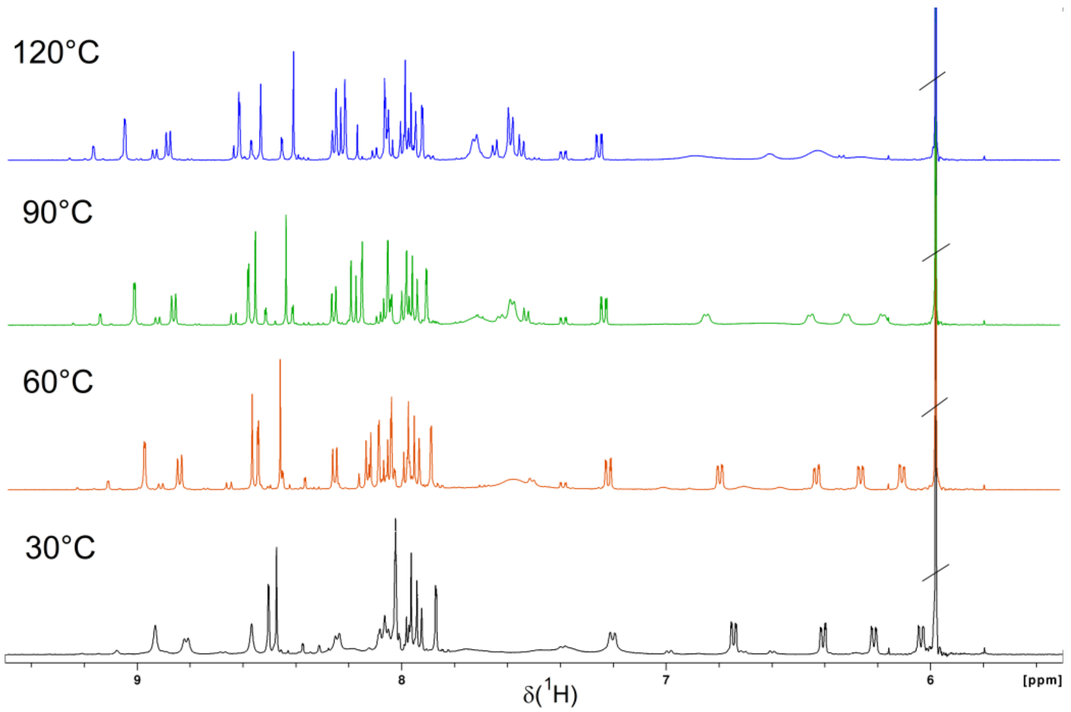

Figure S39. Variable temperature ^{1}H NMR spectra (region) of **5b** (solvent: $C_2D_2Cl_4$).

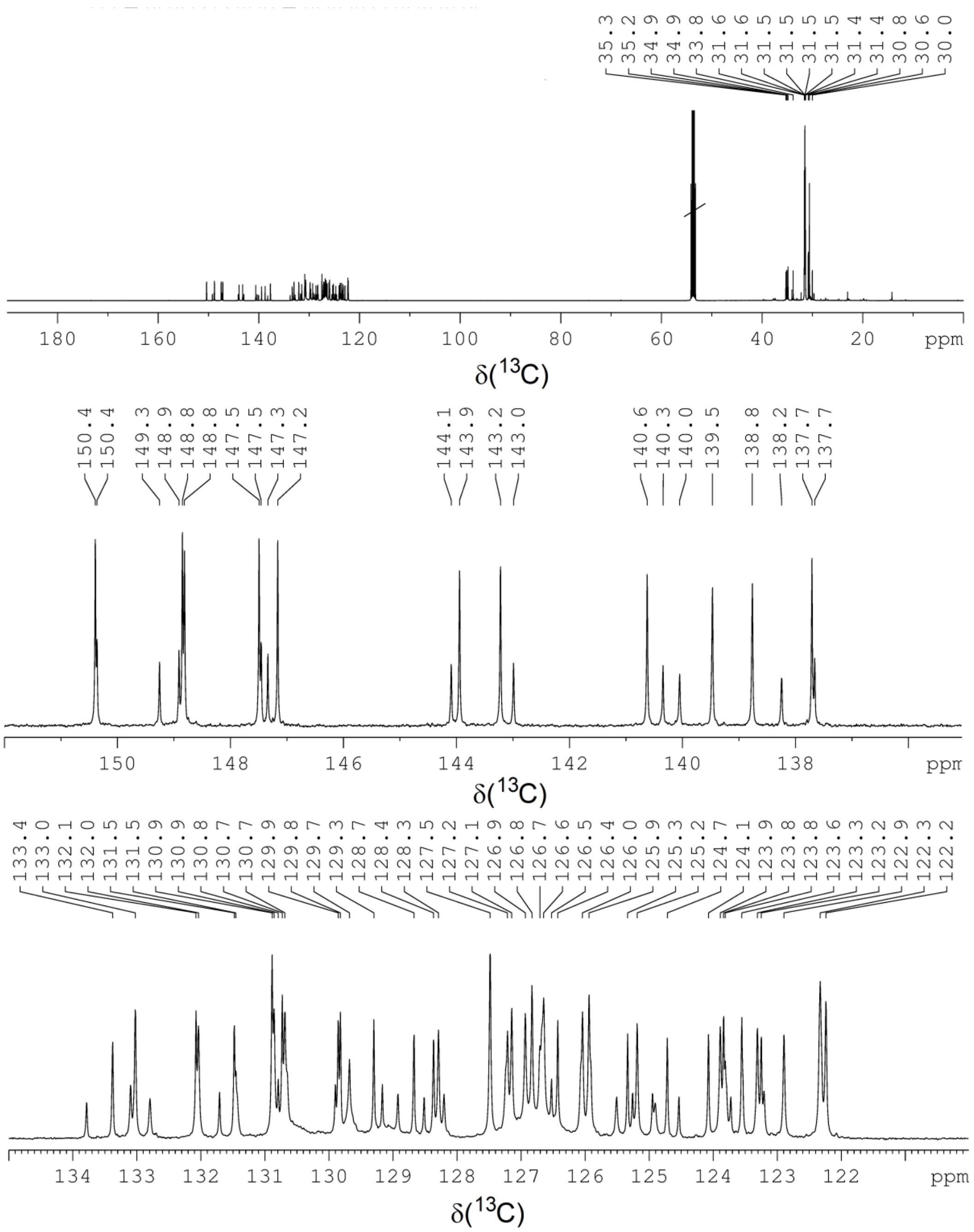

Figure S40. ^{13}C NMR spectrum of **5b** – overview and regions (solvent: CD_2Cl_2).

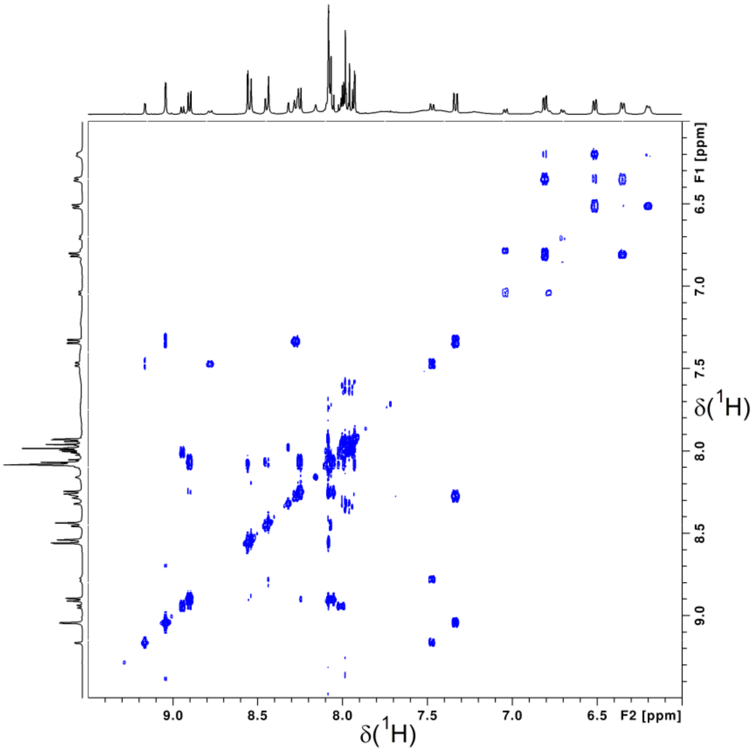

Figure S41. COSY spectrum (region) of **5b** (solvent: CD_2Cl_2).

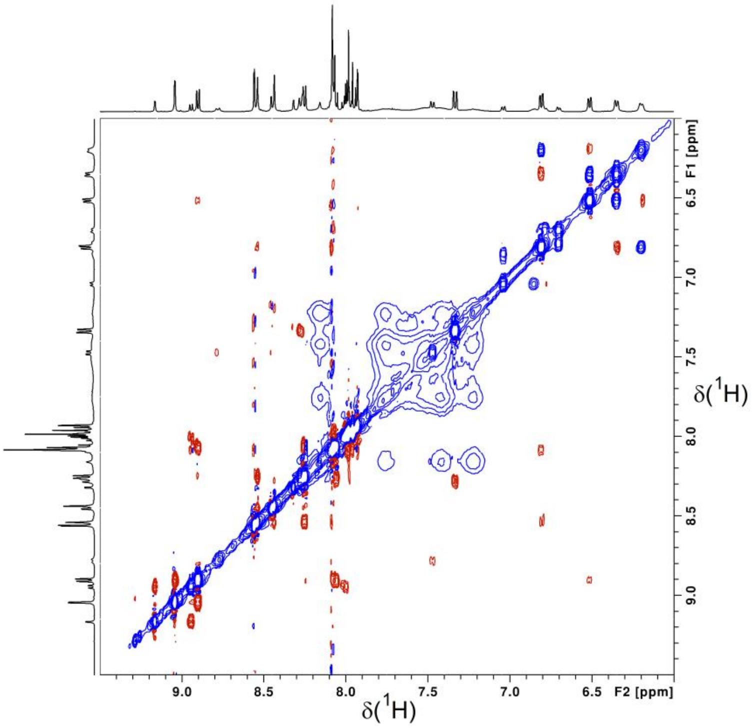

Figure S42. ROESY spectrum (region) of **5b** (solvent: CD_2Cl_2). The red off-diagonal peaks indicate ROESY correlations. The blue off-diagonal peaks indicate exchange correlations.

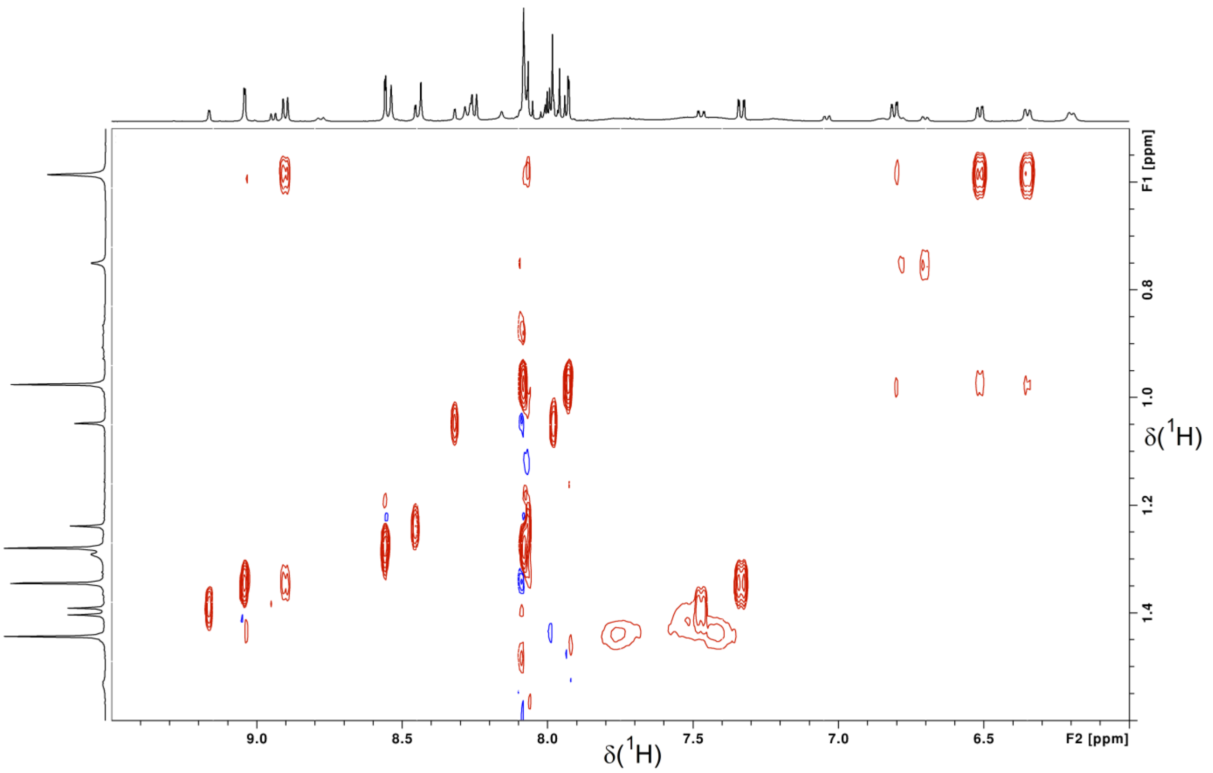

Figure S43. ROESY spectrum (region) of **5b** showing ROESY correlation peaks between tert.-butyl group signals and signals of aromatic protons (solvent: CD_2Cl_2).

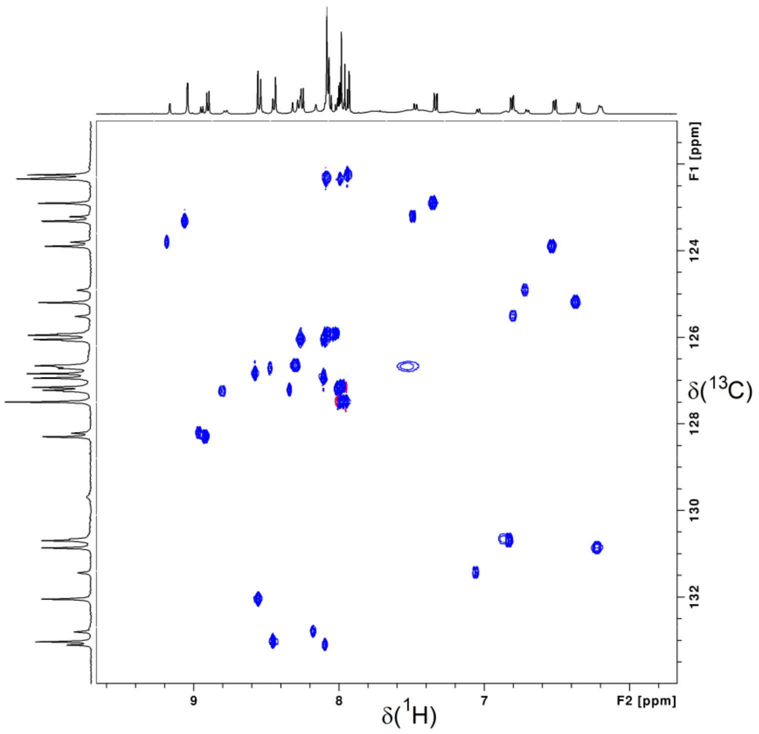

Figure S44. HSQC spectrum (region) of **5b** (solvent: CD_2Cl_2).

8. High-resolution mass spectroscopy (HRMS)

Compound Table

Compound Label	RT	Mass	Abund	Formula	Tgt Mass	Diff (ppm)
Cpd 1: C40 H22	1.221	502.17181	147229	C40 H22	502.17215	-0.68

MS Zoomed Spectrum

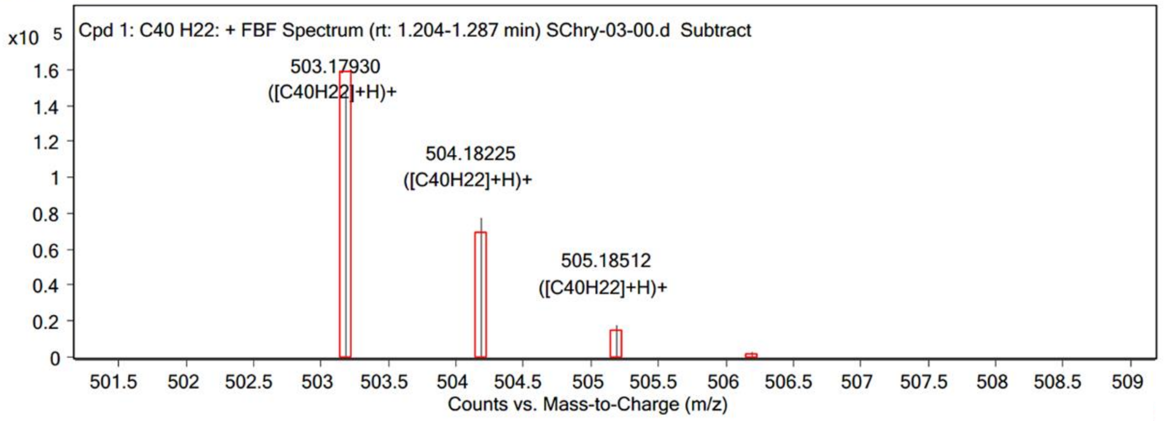

MS Spectrum Peak List

m/z	z	Abund	Formula	Ion
503.1793	1	147228.64	C40H22	(M+H)+
504.18225	1	77435.48	C40H22	(M+H)+
505.18512	1	17762.25	C40H22	(M+H)+
506.18799	1	2811.21	C40H22	(M+H)+

Figure S45. HR-APCI mass spectrum of compound **3a**.

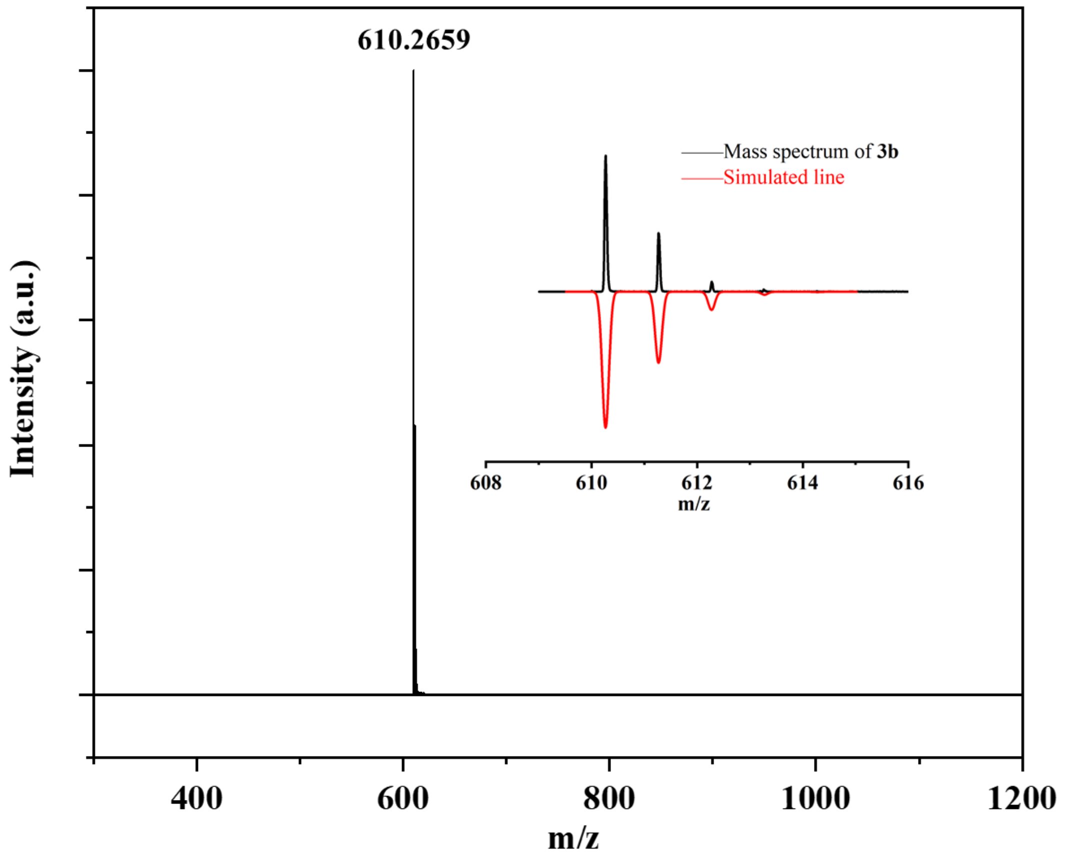

Figure S46. HR-MALDI-TOF mass spectrum of compound **3b**.

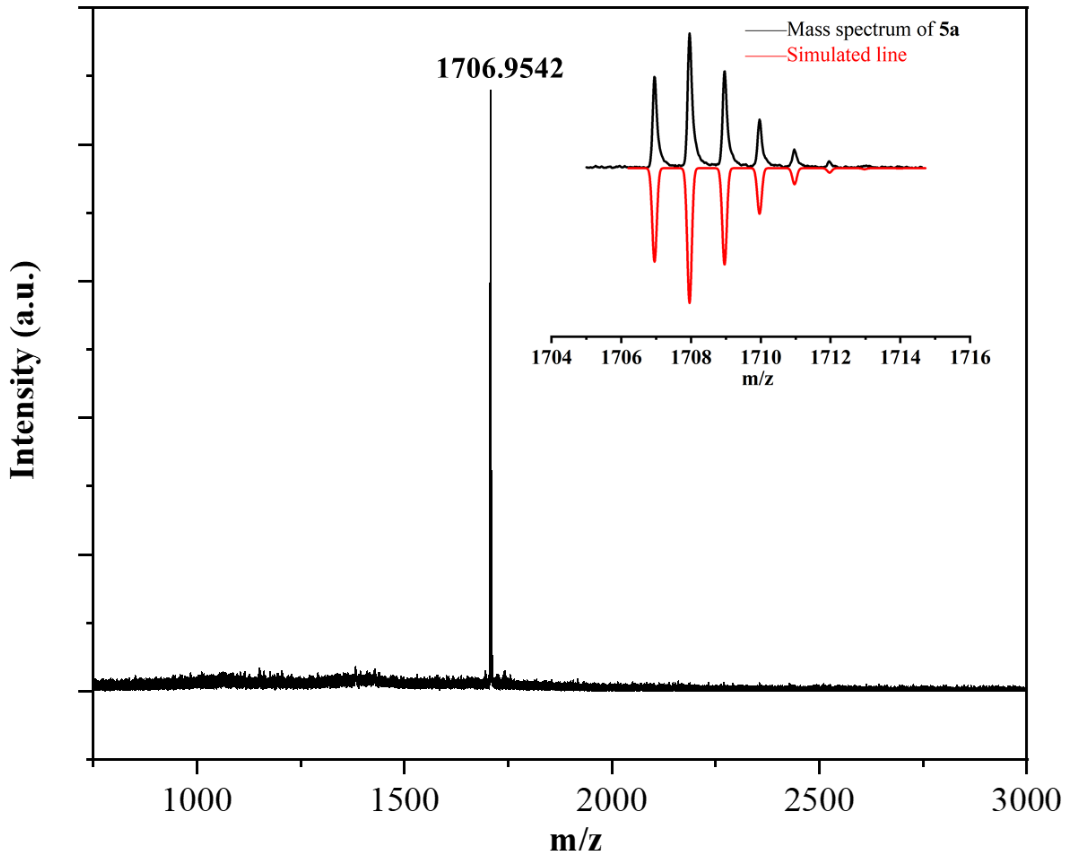

Figure S47. HR-MALDI-TOF mass spectrum of compound **5a**.

9. References

[1] S. Hayashi, K. Hayamizu, *Bull. Chem. Soc. Jpn.* **1991**, *64* (2), 685.

[2] F. Xu, C. Yu, A. Tries, H. Zhang, M. Kläui, K. Basse, M. R. Hansen, N. Bilbao, M. Bonn, H. I. Wang, Y. Mai, *J. Am. Chem. Soc.* **2019**, *141* (28), 10972.

[3] K. Saalwächter, F. Lange, K. Matyjaszewski, C.-F. Huang, R. Graf, *J. Magn. Reson.* **2011**, *212* (1), 204.

[4] D. Massiot, F. Fayon, M. Capron, I. King, S. Le Calvé, B. Alonso, J. O. Durand, B. Bujoli, Z. Gan, G. Hoatson, *Magn. Reson. Chem.* **2002**, *40* (1), 70.

[5] J. Liu, B.-W. Li, Y.-Z. Tan, A. Giannakopoulos, C. Sanchez-Sanchez, D. Beljonne, P. Ruffieux, R. Fasel, X. Feng, K. Müllen, *J. Am. Chem. Soc.* **2015**, *137* (18), 6097.

[6] J. Ma, K. Zhang, K. S. Schellhammer, Y. Fu, H. Komber, C. Xu, A. A. Popov, F. Hennersdorf, J. J. Weigand, S. Zhou, W. Pisula, F. Ortmann, R. Berger, J. Liu, X. Feng, *Chem. Sci.* **2019**, *10* (14), 4025.

[7] a) T. Yamato, A. Miyazawa, M. Tashiro, *Chem. Ber.* **1993**, *126* (11), 2505; b) J. Hu, D. Zhang, F. W. Harris, *J. Org. Chem.* **2005**, *70* (2), 707.

[8] a) A. Narita, X. Feng, Y. Hernandez, S. A. Jensen, M. Bonn, H. Yang, I. A. Verzhbitskiy, C. Casiraghi, M. R. Hansen, A. H. Koch, G. Fytas, O. Ivasenko, B. Li, M. K. S, T. Balandina, S. Mahesh, S. De Feyter, K. Müllen, *Nat. Chem.* **2014**, *6* (2), 126; b) A. Narita, I. A. Verzhbitskiy, W. Frederickx, K. S. Mali, S. A. Jensen, M. R. Hansen, M. Bonn, S. De Feyter, C. Casiraghi, X. Feng, K. Müllen, *ACS nano* **2014**, *8* (11), 11622.

[9] Gaussian 16, Revision A.01, Frisch, M. J.; Trucks, G. W.; Schlegel, H. B.; Scuseria, G. E.; Robb, M. A.; Cheeseman, J. R.; Scalmani, G.; Barone, V.; Petersson, G. A.; Nakatsuji, H.; Li, X.; Caricato, M.; Marenich, A. V.; Bloino, J.; Janesko, B. G.; Gomperts, R.; Mennucci, B.; Hratchian, H. P.; Ortiz, J. V.; Izmaylov, A. F.; Sonnenberg, J. L.; Williams-Young, D.; Ding, F.; Lipparini, F.; Egidi, F.; Goings, J.; Peng, B.; Petrone, A.; Henderson, T.; Ranasinghe, D.; Zakrzewski, V. G.; Gao, J.; Rega, N.; Zheng, G.; Liang, W.; Hada, M.; Ehara, M.; Toyota, K.; Fukuda, R.; Hasegawa, J.; Ishida, M.; Nakajima, T.; Honda, Y.; Kitao, O.; Nakai, H.; Vreven, T.; Throssell, K.; Montgomery, J. A., Jr.; Peralta, J. E.; Ogliaro, F.; Bearpark, M. J.; Heyd, J. J.; Brothers, E. N.; Kudin, K. N.; Staroverov, V. N.; Keith, T. A.; Kobayashi, R.; Normand, J.; Raghavachari, K.; Rendell, A. P.; Burant, J. C.; Iyengar, S. S.; Tomasi, J.; Cossi, M.; Millam, J. M.; Klene, M.; Adamo, C.; Cammi, R.; Ochterski, J. W.; Martin, R. L.; Morokuma, K.; Farkas, O.; Foresman, J. B.; Fox, D. J. Gaussian, Inc., Wallingford CT, **2016**.

[10] Krukau, A. V.; Vydrov, O. A.; Izmaylov, A. F.; Scuseria, G. E. Influence of the exchange screening parameter on the performance of screened hybrid functionals. *J. Chem. Phys.* **2006**, *125*(22), 224106.

[11] Ditchfield, R.; Hehre, W. J.; Pople, J. A. Self-Consistent Molecular Orbital Methods. 9. Extended Gaussian-type basis for molecular-orbital studies of organic molecules. *J. Chem. Phys.* **1971**, *54*(2), 724.

[12] K. Baumgärtner, A. L. Meza Chincha, A. Dreuw, F. Rominger, M. Mastalerz, *Angew. Chem., Int. Ed.* **2016**, *55* (50), 15594.

[13] a) W. Niu, J. Ma, P. Soltani, W. Zheng, F. Liu, A. A. Popov, J. J. Weigand, H. Komber, E. Poliani, C. Casiraghi, J. Droste, M. R. Hansen, S. Osella, D. Beljonne, M. Bonn, H. I. Wang, X. Feng, J. Liu, Y. Mai, *J. Am. Chem. Soc.* **2020**, *142* (43), 18293; b) X. Wang, J. Ma, W. Zheng, S. Osella, N. Arisnabarreta, J. Droste, G. Serra, O. Ivasenko, A. Lucotti, B. David, M. Bonn, X. Liu, M. R. Hansen, M. Tommasini, S. De Feyter, J. Liu, H. I. Wang, X. Feng, *J. Am. Chem. Soc.* **2021**, *144* (1), 228.

[14] a) R. Ulbricht, E. Hendry, J. Shan, T. F. Heinz, M. Bonn, *Rev. Mod. Phys.* **2011**, *83* (2), 543; b) S. A. Jensen, R. Ulbricht, A. Narita, X. Feng, K. Müllen, T. Hertel, D. Turchinovich, M. Bonn, *Nano Lett.* **2013**, *13* (12), 5925; c) I. Ivanov, Y. Hu, S. Osella, U. Beser, H. I. Wang, D. Beljonne, A. Narita, K. Müllen, D. Turchinovich, M. Bonn, *J. Am. Chem. Soc.* **2017**, *139* (23), 7982; d) R. Momper, H. Zhang, S. Chen, H. Halim, E. Johannes, S. Yordanov, D. Braga, B. Blülle, D. Doblas, T. Kraus, *Nano Lett.* **2020**, *20* (6), 4102; e) A. Tries, S. Osella, P. Zhang, F. Xu, C. Ramanan, M. Kläui, Y. Mai, D. Beljonne, H. I. Wang, *Nano Lett.* **2020**, *20* (5), 2993.
